# Multiple Citation Indicators and Their Composite across Scientific Disciplines

**DOI:** 10.1371/journal.pbio.1002501

**Published:** 2016-07-01

**Authors:** John P.A. Ioannidis, Richard Klavans, Kevin W. Boyack

**Affiliations:** 1 Meta-Research Innovation Center at Stanford (METRICS), Stanford University, Stanford, California, United States of America; 2 Stanford Prevention Research Center, Department of Medicine and Department of Health Research and Policy, Stanford University School of Medicine, Stanford, California, United States of America; 3 Department of Statistics, Stanford University School of Humanities and Sciences, Stanford, California, United States of America; 4 SciTech Strategies, Inc., Wayne, Pennsylvania, United States of America; 5 SciTech Strategies, Inc., Albuquerque, New Mexico, United States of America; Northwestern University, UNITED STATES

## Abstract

Many fields face an increasing prevalence of multi-authorship, and this poses challenges in assessing citation metrics. Here, we explore multiple citation indicators that address total impact (number of citations, Hirsch H index [H]), co-authorship adjustment (Schreiber Hm index [Hm]), and author order (total citations to papers as single; single or first; or single, first, or last author). We demonstrate the correlation patterns between these indicators across 84,116 scientists (those among the top 30,000 for impact in a single year [2013] in at least one of these indicators) and separately across 12 scientific fields. Correlation patterns vary across these 12 fields. In physics, total citations are highly negatively correlated with indicators of co-authorship adjustment and of author order, while in other sciences the negative correlation is seen only for total citation impact and citations to papers as single author. We propose a composite score that sums standardized values of these six log-transformed indicators. Of the 1,000 top-ranked scientists with the composite score, only 322 are in the top 1,000 based on total citations. Many Nobel laureates and other extremely influential scientists rank among the top-1,000 with the composite indicator, but would rank much lower based on total citations. Conversely, many of the top 1,000 authors on total citations have had no single/first/last-authored cited paper. More Nobel laureates of 2011–2015 are among the top authors when authors are ranked by the composite score than by total citations, H index, or Hm index; 40/47 of these laureates are among the top 30,000 by at least one of the six indicators. We also explore the sensitivity of indicators to self-citation and alphabetic ordering of authors in papers across different scientific fields. Multiple indicators and their composite may give a more comprehensive picture of impact, although no citation indicator, single or composite, can be expected to select all the best scientists.

## Abstract 2

Citation indicators addressing total impact, co-authorship, and author positions offer complementary insights about impact. This article shows that a composite score including six citation indicators identifies extremely influential scientists better than single indicators.

## Author Summary

Multiple citation indicators are used in science and scientific evaluation. With an increasing proportion of papers co-authored by many researchers, it is important to account for the relative contributions of different co-authors. We explored multiple citation indicators that address total impact, co-authorship adjustment, and author order (in particular, single, first, or last position authorships, since these positions suggest pivotal contributions to the work). We evaluated the top 30,000 scientists in 2013 based on each of six citation indicators (84,116 total scientists assessed) and also developed a composite score that combines the six indicators. Different scientists populated the top ranks when different indicators were used. Many Nobel laureates and other influential scientists rank among the top 1,000 with the composite indicator, but rank much lower based on total citations. Conversely, many of the top 1,000 authors on total citations had no single/first/last-authored cited paper. More Nobel laureates are among the top authors when authors are ranked by the composite score than by single indicators. Multiple indicators and their composite give a more comprehensive picture of impact, although no method can pick all the best scientists.

## Introduction

Citation indicators have become very popular in appraising the published work of scientists. However, they are also known to have major caveats that can lead to misleading conclusions [1]. To confuse matters more, many different citation indicators currently compete for prominence. Besides the total citation count, perhaps the most popular indicator is the Hirsch H index (H) [2], and there have been long debates about its relative merits and drawbacks [3–6]. Moreover, it is increasingly recognized that in most scientific fields there is an increasing number of co-authors per paper [7–9]. Some fields, such as particle physics, are operating routinely with large-scale collaborations with hundreds of authors. Many fields in biology, genomics, epidemiology, and medicine also have many papers with large numbers of authors. Therefore, citation indicators have been proposed that account for co-authorship [10–12]. One may also want to separate citations according to whether they come from single-authored work or whether the scientist has a leading role as suggested by authorship placement, e.g., first or last author. Of course, with the exception of single-authored work, the relative contribution of authors cannot be fully ascertained from authorship position alone. However, empirical surveys suggest that, except when listing of authorship is alphabetic, first and last authors have typically had the greatest contributions in a multi-authored paper [13–16].

There are numerous surveys that investigate one or a few of the proposed citation indicators in samples of scientists limited to specific scientific disciplines. It would be more useful, however, to assess a large number of the most prominent citation indicators across all the tens of thousands of scientists with substantial citation impact across all scientific fields. This could help us understand to what extent different citation indicators are correlated with each other or offer independent information. Furthermore, one could assess whether these correlation patterns differ across different scientific fields. Finally, one could generate composite indicators that incorporate information from multiple citation indicators. A composite may be more appropriate, because it would not be that much influenced by extreme values in only one indicator.

Here, we performed such an evaluation of 84,116 influential scientists across all scientific disciplines around the world. For further details, see Methods. All citation indicators, unless specified otherwise, refer to the citation impact of the scientists’ work in the calendar year 2013. Scopus contains very complete information, including reference lists, for documents published since 1996. Coverage for documents published before 1996 is somewhat (~15%) less, and Scopus is only now adding reference lists for documents published from 1970 to 1995. This limits our ability to assess the full citation profile of a scientist over his/her entire career for careers that were initiated prior to 1996. Perhaps more importantly, one would like to focus on the recent citation impact of a scientist, as this may be more reflective of both his/her current visibility and his/her future citation rate (e.g., the number of citations in a given year is the strongest predictor of citations in future years [17]) and perhaps also the potential for continuing to perform influential work in the near future. However, in principle, the same approach as we outline in this manuscript can also be applied in evaluating citation metrics of scientists covering multiple years (if this is considered desirable for whatever reason) or even the entire career (if databases have coverage for the respective time span).

## Results

### Citation Indicators and Selection Thresholds for Top-Cited Scientists

We considered the following six indicators, which may be informative about citation impact in a monotonic fashion (the higher the better): total number of citations received in 2013 (NC), total number of citations received in 2013 to papers for which the scientist is single author (NS), total number of citations received in 2013 to papers for which the scientist is single or first author (NSF), total number of citations received in 2013 to papers for which the scientist is single, first, or last author (NSFL), Hirsch H index for the citations received in 2013 (H), and Schreiber co-authorship adjusted Hm index for the citations received in 2013 (Hm). We also recorded the numbers of papers (Scopus-indexed scholarly works) by each scientist published up to and including 2013, and the respective number of citations per paper in 2013; these two metrics are not necessarily monotonically related with higher impact, and they are inversely related among themselves by definition.

We identified the scientists with the top 30,000 values in each of the six different monotonic indicators. The thresholds corresponding to the 30,000th ranked scientist for each indicator are 925 citations for NC, H = 14, Hm = 6.239, NS = 31, NSF = 158, and NSFL = 348.

Using these thresholds, we identified a total of 84,565 unique author identifiers, of which 278 (of the 10,000 with the largest numbers of publications) were excluded upon scrutiny since they seemed to reflect cases of polysemy (multiple different scientists with the same name merged under the same Scopus author ID). Moreover, some scientists in the list had multiple author identifiers. After merging these same-author identifiers, a total of 84,116 scientists remained. Comparison against funding databases has also shown that Scopus identifiers have very high precision for matching to specific single authors [18]; therefore, we expect that these 84,116 names reflect single authors with high accuracy.

### Correlation between Citation Indicators

For analysis, each of the six indicators listed above was log-transformed. Table 1 shows Pearson correlations between these log-transformed indicators across all 84,116 scientists. As shown in Table 1, the correlation of NC and H is strong (*r* = 0.88), suggesting that most of the time, with some exceptions, total citations and H-index gave the same impression about the ranking of a scientist. The exceptions occurred mostly because of scientists who had (co)-authored only a few papers with very large citation impact. Exclusion of 8,481 scientists with fewer than 30 papers indexed in Scopus resulted in an even stronger correlation (*r* = 0.91) between NC and H.

**Table 1:** Correlations between various citation indicators and other metrics across all 84,116 scientists.

	***NC***	***H***	***Hm***	***NS***	***NSF***	***NSFL***	***C***	***NP***	***Cpp***
***NC***	1.00								
***H***	0.88	1.00							
***Hm***	0.00	0.19	1.00						
***NS***	-0.43	-0.34	0.42	1.00					
***NSF***	-0.22	-0.12	0.72	0.45	1.00				
***NSFL***	-0.04	0.04	0.88	0.34	0.83	1.00			
***C***	0.11	0.25	0.92	0.54	0.83	0.89	1.00		
***NP***	0.55	0.47	0.14	-0.11	-0.11	0.06	0.17	1.00	
***Cpp***	0.09	-0.14	-0.22	-0.12	-0.13	-0.13	-0.19	-0.12	1.00

All 95% confidence intervals have width <0.01 given the large numbers analyzed. Abbreviations: NC, total citations; H: Hirsch H index; Hm: Schreiber Hm index; NS: total citations to papers for which the scientist is single author; NSF: total citations to papers for which the scientist is single or first author; NSFL: total citations to papers for which the scientist is single, first, or last author; C: composite citation indicator; NP: number of papers; Cpp: citations per paper

Conversely, there was no correlation between the total number of citations and the co-authorship adjusted Hm (*r* = 0.00), and there was a negative correlation between total number of citations and each of the three author-order-based citation indicators (*r* = -0.43, *r* = -0.22, and *r* = -0.04 for NS, NSF, and NSFL, respectively, *p* < 0.001 for all). Some of the most-cited authors (in terms of citation volume, i.e., total citations or H index) had published very few or even no influential papers as single, first, or last authors. For example, of the 100 most-cited authors according to total citations (NC = 7,457–19,245 Scopus citations received in 2013), almost half of them (*n* = 48) had received only 0–20 citations to papers or other scholarly documents for which they were the single author, and 15 of them did not have even a single cited single-authored paper or other scholarly document. Moreover, of the 100 most-cited authors according to total citations, 12 had received fewer than 100 citations in papers for which they were the single or first author, and 11 had received fewer than 1,000 citations to all papers for which they were single, first, or last author. Thus, selection of top scientists based on total citations only may include many scientists without a leading, key contribution to the published work.

Indicators of citation volume (total number of citations and H) were somewhat strongly correlated with the number of papers. However, they had no substantial correlation with the mean number of citations per paper (Table 1). Visual correlations between log-transformed pairs of the six individual indicators and composite indicator are shown in S1 Fig.

### Field-Specific Analyses

**Fig 1:**
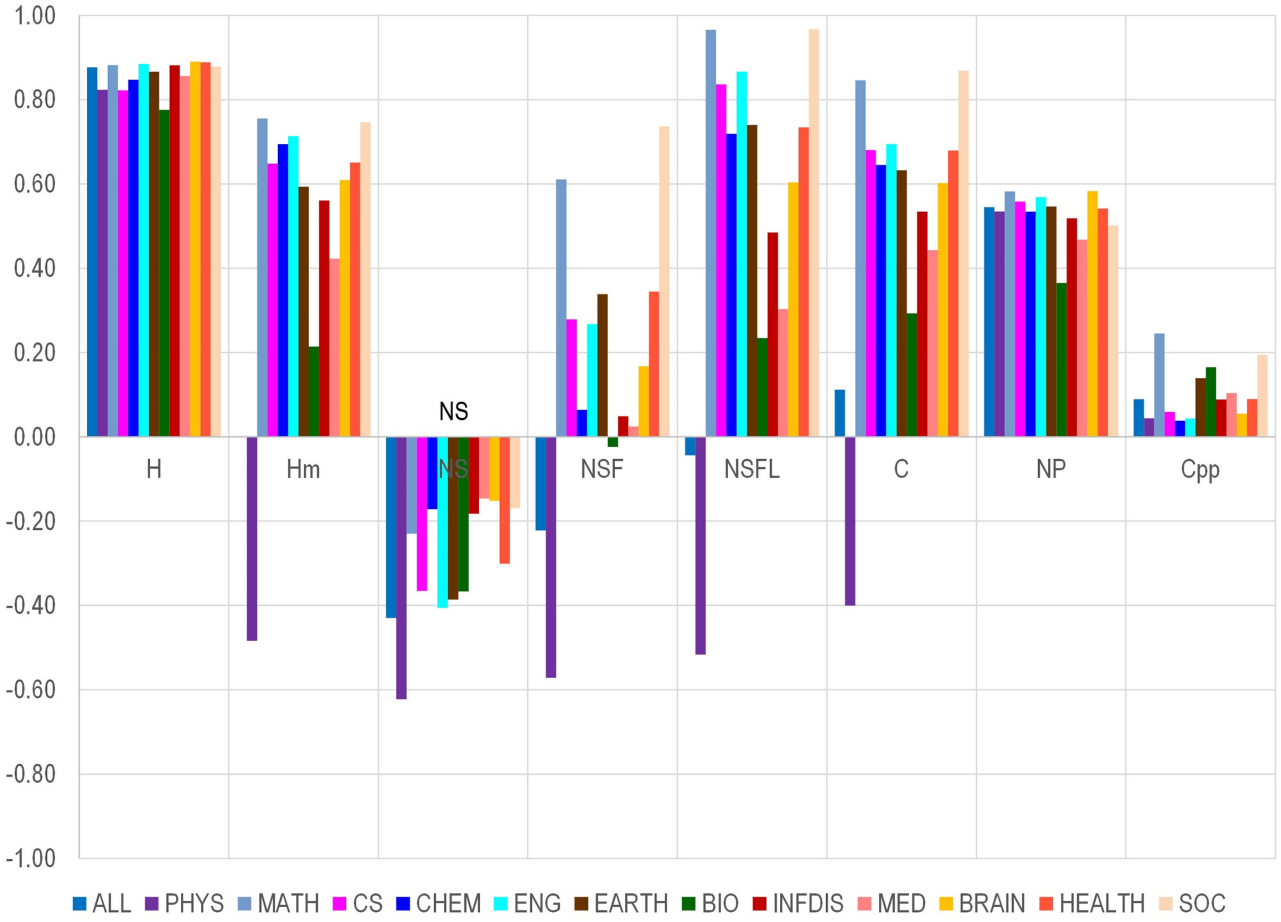
Correlation between number of citations and various citation indicators and other metrics in each of 12 different scientific fields. Abbreviations: PHYS, physics; MATH, mathematics; CS, computer science; CHEM, chemistry; ENG, engineering; EARTH, earth sciences; BIO, biology/biotechnology. INFDIS, infectious disease; MED, medicine; BRAIN, brain research; HEALTH, health sciences; SOC, social sciences. No data are shown on humanities, for which there are too few papers and too few citations in Scopus to allow meaningful analysis.

Each of the published items indexed in Scopus was allocated into one of 12 fields (physics, mathematics, computer science, chemistry, earth sciences, engineering, biology/biotechnology, infectious disease, medicine, brain research, health sciences, social sciences) that have been defined in previous work [19,20]. Then, we allocated each of the 84,116 scientists to the scientific discipline that had the largest share of his/her published work indexed in Scopus. We separately calculated the correlations of NC with all other indicators in each scientific discipline that included at least 1,000 scientists. As shown in Fig 1, the correlation between NC and H was always strong across all disciplines (*r* = 0.776–0.890). NC was negatively correlated with NS in all disciplines and was most strongly negatively in physics. Physics, which has the strongest tradition of extremely multi-authored papers, stands out from the other disciplines in that NC was negatively correlated with all four author-adjusted or author-order indicators (NS, NSF, NSFL, and Hm index). Excluding physics, the correlation of NC with the co-authorship-adjusted Hm was substantial, although quite variable across disciplines (*r* = 0.21–0.76) and always smaller than the correlation between NC and H. NC correlated positively with NSF in all disciplines but biology and physics, with correlations ranging from weak (medicine, *r* = 0.02) to strong (social sciences, *r* = 0.74). Correlations of NC with NSFL were much stronger, above 0.7 for all disciplines except biology, infectious disease, and medicine. The correlation between NC and the number of papers varied moderately across disciplines (from *r* = 0.37 in biology to *r* = 0.58 in brain sciences and mathematics). There were no substantial correlations between NC and the number of citations per paper in any discipline, except for mathematics (*r* = 0.25).

### Composite Citation Indicator

This picture suggests that citation indicators may correlate to a variable extent with each other, and this pattern may also fluctuate from one field to another. Examining all of these indicators may give more granular information about the impact of a scientist than a single indicator alone. Moreover, one possibility is to combine all these indicators into a composite indicator that draws information from each and all of them. Accordingly, we standardized each of the six log-transformed citation indicators (NC, H, Hm, NS, NSF, NSFL), giving each a standardized value from 0 to 1, where 1 is given to the scientist with the highest raw value for the respective indicator. We then summed the six standardized indicators and generated a composite index C. Factor analysis shows that the six indicators separate into two factors: one associated with bulk impact (NC and H) and the other associated with author order and co-authorship-adjusted impact (Hm, NS, NSF, and NSFL). These two factors are roughly balanced with each other in the composite indicator.

S1 Table shows the proportion of the scientists categorized in each of the 12 key fields among the top- 1,000, top 3,000, top 10,000, top 20,000, and top 30,000 based on the composite score and among all 84,116 scientists analyzed. In theory, a scientist who has values just below the top 30,000 in each and every of the six indicators included in the composite could still have the same composite score as the current rank 14,150. Therefore, the list of the top 14,150 scientists is all-inclusive. Details on all 84,116 scientists are available in S1 Data. For convenience, S2 Table shows the indicators for the 1,000 scientists with the highest composite values across all scientific disciplines. Of those, 167 originally had split records (their papers had been classified by Scopus under two or three different author IDs), which were merged prior to analysis.

### Top-Ranked Scientists with the Composite Score versus with Total Citations

Of the 1,000 top-ranked scientists according to the composite score, only 322 were also among the 1,000 top-ranked scientists based on total citations, i.e., had received ≥3,933 citations in 2013. A large proportion of the 1,000 top-ranked scientists based on the composite score would have been ranked much lower if only total citations had been considered. We examined information (retrieved online with name searches) on the curricula vitae and accomplishments of the 120 scientists who had received only 853–1,703 total citations in 2013. All of them were deemed to be highly visible scientists, although one had been involved in alleged misconduct. Among these 120 scientists who would have otherwise ranked relatively low based on total citations alone, we identified 9 Nobel prize winners (Elizabeth Blackburn, NC = 1,697; Richard R. Schrock, NC = 1,684; Eugene F. Fama, NC = 1,672; James J. Heckman, NC = 1,598; Pierre-Giles De Gennes, NC = 1,495; Steven Weinberg, NC = 1,436; Frank Wilczek, NC = 1,408; Harald zur Hausen, NC = 1,324; Philip W. Anderson, NC = 1,158). We also found among these 120 scientists many other science stars, such as Stephen W. Hawking, two MacArthur fellows (David Montgomery, Nancy Moran), and winners of many other leading awards, such as Breakthrough, Shaw, Lasker, and Darwin prizes, as well as dozens of members of highly reputable academies.

Conversely, of the 1,000 top-ranked scientists based on total citations alone, 122 have not had even a single citation to a paper for which they were single, first, or last author, and 258 had no more than 20 such citations. We randomly selected 20 scientists who were among the 1,000 top-ranked for total citations, but did not have any citations to a paper for which they were single, first, or last author. Of those 20, 11 were physics professors (full professor *n* = 7, associate professor *n* = 4) working on collaborative research, 3 were research scientists (staff or other non-faculty), 2 were postdoctoral fellows, 1 was a web application developer, and for 3 of them we could not find sufficient information online on their rank and affiliation.

### Capturing Nobel Laureates of 2011–2015 with Different Metrics

Table 2 shows, for each of the Nobel laureates of the years 2011–2015 in physics, chemistry, physiology/medicine, and economics, whether he/she belongs to the top 14,150 scientists according to the composite score and, similarly, to the top 14,150 according to total citations, H index, or Hm index (as above, we know that the ranking is inclusive for the top 14,150 ranked scientists). It also shows whether they are among the top 30,000 scientists according to any of the six metrics included in the composite score.

**Table 2:** Capturing Nobel laureates of 2011–2015 by top rank in 2013 with different metrics.

Nobel laureate	NC (top 14,150)	H (top 14,150)	Hm (top 14,150)	Composite (top 14,150)	Any of the six metrics (top 30,000)
Takaaki Kajita	No	No	No	No	No
Arthur B. Mcdonald	No	No	No	No	No
Isamu Akasaki	No	No	No	No	Yes
Hiroshi Amano	No	No	No	No	No
Shuji Nakamura	Yes	Yes	Yes	Yes	Yes
Francois Englert	No	No	No	No	Yes
Peter W. Higgs	No	No	No	No	Yes
Serge Haroche	No	No	No	No	Yes
David J. Wineland	Yes	Yes	Yes	Yes	Yes
Saul Perlmutter	Yes	No	No	Yes	Yes
Brian P. Schmidt	Yes	No	No	No	Yes
Adam G. Riess	Yes	Yes	No	Yes	Yes
Tomas Lindahl	No	Yes	Yes	Yes	Yes
Paul Modrich	No	No	Yes	Yes	Yes
Aziz Sancar	No	No	Yes	Yes	Yes
Eric Betzig	No	No	No	Yes	Yes
Stefan W. Hell	Yes	Yes	Yes	Yes	Yes
William E. Moerner	No	Yes	Yes	Yes	Yes
Martin Karplus	Yes	Yes	Yes	Yes	Yes
Michael Levitt	No	No	Yes	Yes	Yes
Arieh Warshel	Yes	No	Yes	Yes	Yes
Robert J. Lefkowitz	Yes	Yes	Yes	Yes	Yes
Brian K. Kobilka	Yes	Yes	Yes	Yes	Yes
Dan Shechtman	No	No	No	No	Yes
William C. Campbell	No	No	No	No	No
Satoshi Omura	No	No	No	Yes	Yes
Youyou Tu	No	No	No	No	No
John O’Keefe	No	No	Yes	Yes	Yes
May-Britt Moser	No	Yes	Yes	Yes	Yes
Edvard I. Moser	No	Yes	Yes	Yes	Yes
James E. Rothman	No	Yes	Yes	Yes	Yes
Randy W. Schekman	No	No	Yes	No	Yes
Thomas C. Sudhof	Yes	Yes	Yes	Yes	Yes
John B. Gurdon	No	No	Yes	Yes	Yes
Shinya Yamanaka	Yes	Yes	Yes	Yes	Yes
Bruce A. Beutler	Yes	Yes	Yes	Yes	Yes
Jules A. Hoffmann	No	Yes	Yes	Yes	Yes
Ralph M. Steinman	Yes	Yes	Yes	Yes	Yes
Angus Deaton	No	No	Yes	Yes	Yes
Jean Tirole	No	Yes	Yes	Yes	Yes
Eugene F. Fama	Yes	Yes	Yes	Yes	Yes
Lars Peter Hansen	No	No	No	No	No
Robert J. Shiller	No	No	No	No	Yes
Alvin E. Roth	No	No	Yes	Yes	Yes
Lloyd S. Shapley	No	No	No	No	Yes
Thomas J. Sargent	No	No	No	No	No
Christopher A. Sims	No	No	Yes	Yes	Yes

As shown, the composite score captures 31 of the 47 laureates, as opposed to only 15 captured by the total citations, 18 by the H index, and 26 by the Hm index. A total of 40 of the 47 laureates were in the top 30,000 scientists in 2013, according to at least one of the six metrics included in the composite score. The seven Nobel laureates who were not captured by any of the six indicators are useful to scrutinize in order to understand why these indicators failed. The seven exceptions included two economists (Lars Peter Hansen and Thomas J. Sargent) for whom Scopus had poor coverage of citations for their work (they are both highly cited in Google Scholar, which provides >10-fold more citations for them than Scopus); two scientists with a major contribution in the development of important antiparasitic drugs (Williams C. Campbell and Youyou Tu), whose work is not that highly cited; and three physicists (Takaaki Kajita, Arthur B. McDonald, Hiroshi Amano) who are very highly cited, but their citations peaked >10 y earlier (moreover, the most-cited papers for Kajita and McDonald had alphabetic authorship).

### Number of Papers and Citations per Paper

In contrast to the six indicators that are included in the composite indicator, for which higher values are better than lower values, the number of papers and the citations per paper are not necessarily better when they are higher. In fact, by definition, these two metrics are inversely related. Excellent scientists may publish few papers or many papers, and this will respectively increase or decrease their citations per paper. There was tremendous variation in these two metrics across the 84,116 scientists: the number of papers varied from 1 to 2,533, and citations per paper varied from 0.08 to 5318. Even among the top 1,000 scientists with the highest composite score, the number of papers varied from 26 to 2,533, and the citations per paper varied from 1.55 to 288.9.

**Fig 2:**
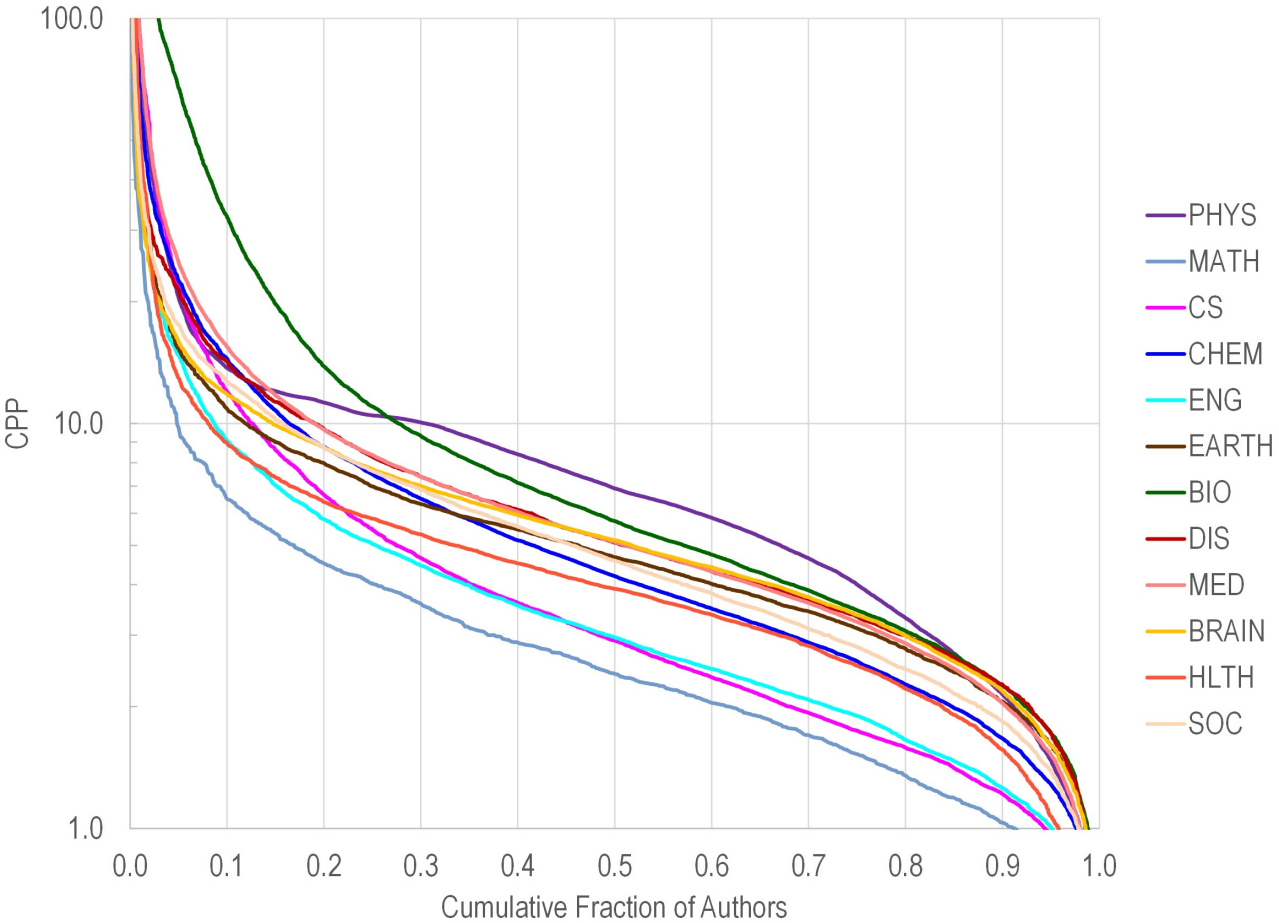
Cumulative proportion of scientists (among total *n* = 84,116) with citations per paper greater than a given number. Data are shown separately for each of 12 scientific disciplines. Scientific discipline abbreviations are the same as in Fig 1.

Fig 2 shows the distribution of citations per paper for authors in various fields. As shown, for most fields, a minority of scientists had extremely high values, and another minority had extremely low values. Perhaps one could wish for investigators to avoid having a low or very low number of citations per paper (and this might also be tailored according to field), while the exact value above that threshold would not make a difference. For example, values below 5 were seen in 42,984 of the 84,116 (51%) scientists, but in only 145 of the 1,000 with the highest composite C scores.

### Impact of Self-Citations

**Fig 3:**
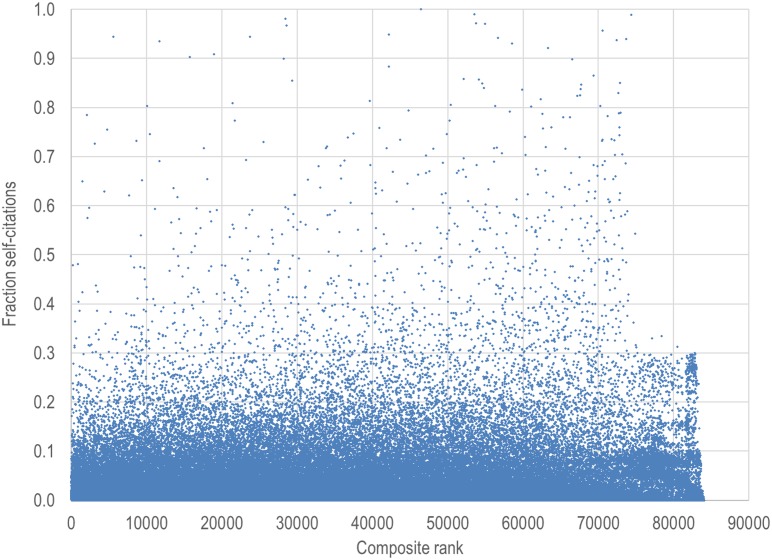
Percentage of self-citations for each of the 84,116 analyzed scientists.

Fig 3 shows the percentage of self-citations for each of the analyzed scientists. Among the 84,116 scientists, the mean was 5.5% and the median was 3.3% (interquartile range, 0.7% to 7.4%). Among the top 1,000 scientists based on the composite score, the mean was 4.2% (median 3.0%, interquartile range 1.3% to 5.4%). The proportion of self-citations exceeded 30% for only 1,206 of the 84,116 scientists (1.45%). Furthermore, only 7 of the top 1,000 scientists based on the composite score (0.7%) have such an extreme self-citation rate.

### Misallocation of Credit Due to Alphabetic Ordering of Authors

Indicators based on author order, such as NSF and NSFL, would be misleading in papers for which authorship order is selected alphabetically rather than reflecting the relative value of contributions. In general, the proportion of papers that are expected to have alphabetic order of authors is 1:k! (where k is the number of authors), i.e., 100%, 50%, 16.67%, 4.17%, 0.83%, 0.14%, 0.02%, 0.002%, and ~0% for papers with k = 1, 2, 3, 4, 5, 6, 7, 8, and >8 authors, respectively. When the proportion of papers with alphabetic order exceeds 1:k!, the excess provides an estimate of the frequency of papers for which authors are ordered alphabetically, not because this reflects their relative contributions, but because this is a chosen authorship listing strategy. The excess was 0%, 3.73%, 4.04%, 2.25%, 1.19%, 0.65%, 0.48%, 0.38%, and 0.40% for the respective values of k. It was 1.97% when data are considered across all articles and all k.

Table 3 shows the proportion of papers with alphabetic listing of authors for k = 3–5 for each of the 12 main scientific fields (plus humanities). As shown, there was a small excess (<1%) from the expected 1:k! in 9 of the 12 scientific fields and a substantially larger excess for physics, mathematics, social sciences and humanities, with mathematics showing the highest incidence of alphabetic ordering.

**Table 3:** Proportion of papers with authors in alphabetic order in different scientific fields (data are shown for papers with 3, 4, and 5 authors).

	Proportion of papers with alphabetic author order		
	Three authors	Four authors	Five authors
*Expected by chance*	*16*.*67%*	*4*.*17%*	*0*.*83%*
Physics	26.56%	10.37%	4.26%
Mathematics	50.96%	32.79%	23.18%
Computer Science	20.37%	7.50%	3.39%
Chemistry	17.34%	5.02%	1.43%
Engineering	18.01%	5.16%	1.56%
Earth Sciences	18.44%	5.83%	1.58%
Biology/Biotechnology	17.20%	4.77%	1.23%
Infectious Disease	17.27%	4.33%	1.14%
Medicine	16.93%	4.41%	1.06%
Brain Research	17.48%	4.46%	0.98%
Health Sciences	17.89%	4.64%	1.10%
Social Sciences	27.74%	11.21%	4.37%
Humanities	31.36%	14.60%	18.18%

## Discussion

Multiple citation indicators may be considered in appraising scientific impact, including those addressing total impact, co-authorship adjustment, and author order. We have demonstrated the complex correlation patterns between these indicators across 84,116 top scientists and separately across 12 scientific fields. Indicators of total impact and co-authorship/author order may be used in a complementary fashion to generate a composite score. No citation indicator, single or composite, can be expected to select all the best scientists. However, the use of a composite may allow for capturing not only citation influence but also aspects of co-authorship and relative credit in multi-authored papers that currently represent the large majority in most scientific fields.

Perusal of the data on the top 1,000 scientists based on the composite indicator shows that they are generally well-known, leading, high-profile scientists. This applied also to the scientists who were top-ranked based on the composite score, but would have been ranked much lower based on total citation count. For example, many Nobel laureates do not necessarily have the highest number of total citations, but they tend to have a pivotal role as single, first, or last authors of some extremely influential works. The vast majority of Nobel laureates of 2011–2015 ranked at extremely high ranks in 2013 by at least one of the six metrics; the few exceptions were scientists with earlier citation peaks, poor coverage of their field in Scopus, and only two great discoverers without any extremely high citation metrics. Furthermore, the composite score included in its top-most ranks a higher number of the Nobel laureates of 2011–2015 than any other metric alone in their respective top-most ranks. In particular, ranking using a traditional metric such as total citations brought to the top ranks large numbers of scientists without any cited papers as single, first, or last authors. An in-depth evaluation of a sample of them suggested that many of them were collaborators in large projects and others were probably auxiliary researchers. For those for whom we could not find their affiliation and rank online, it is even more unlikely that they would have been leading drivers in the published research.

Of course, it is not possible to ever reach certainty that scientists ranked on the top based on the composite score are more influential than others with modestly lower ranking. There is no gold standard for appraising scientist merit. Nevertheless, the selected top-ranked scientists represent a cohort of highly influential scientists who can be used for other empirical assessments [21]. There can also be some debate about whether co-author/author order components should carry similar, less, or more weight as compared with total citation impact. Different weight and approaches to forming a composite citation indicator may select more collaborative types of scientists or more individual leaders [22]. For example, the Thomson Reuters approach for selecting the most influential scientists does not consider author order and multi-authorship (except for extremely multi-authored papers in physics that are excluded) [23]; as a result, the most influential scientist in the world in 2014 and 2015 according to the Thomson Reuters list is a scientist who has not been the first or last author of any highly cited paper.

The proposed composite indicator does not include the number of papers or the citations per paper. We see no point in rewarding a scientist for publishing more papers [24]. While some promotion or funding systems reward such plain productivity, this can stimulate salami publication of least publishable units and sloppy, low-quality work [25]. As for citations per paper, a wide range of values can be equally acceptable. Some investigators have argued in favor of using citations per paper as an indicator of investigator quality [26]. However, this metric depends a lot on the specific sub-field and numbers of co-authors, and values can be markedly affected by outliers (one or a few extremely cited papers). Moreover, over-emphasis on citations per paper may lead investigators to avoid publication of negative, high-quality results and may make them pursue reporting extreme results and exaggerated narratives that draw attention. Nevertheless, it may be reasonable to ask for investigators to maintain citations per paper above a relatively low given threshold. Such an approach would sufficiently discourage salami publication and the publication of least publishable units without discouraging publication of good studies with negative results.

As we admitted up front, citation indicators have limitations, and they should be used with great caution [1]. They should be complemented with in-depth qualitative appraisal of scientific work. However, citation indicators are also here to stay. Our approach suggests how one can gain insights about their behavior and patterns across science and within specific scientific disciplines. Instead of using only one citation indicator, systematically examining multiple indicators offers a more complete, granular picture. Previous empirical assessments have examined the correlation between certain indicators [27,28], but these have been limited mostly to H (and variants of H), NC, and number of papers (NP), without consideration of co-authorship-adjusted and author order indicators. Availability of large-scale citation data on all publishing scientists may also allow for placing these indicators in the context of the entire global scientific community and the scientific community of large disciplines or even smaller focused sub-disciplines. Here, we worked with data on 80,000+ influential scientists, including the ones who are largely shaping the current scientific literature [29], and we placed emphasis on statistics based on author order, which has rarely been done before. However, one can expand the same principles to the many millions of scientists publishing scientific work, provided that the citation databases used adequately cover the specific scientific field being evaluated; otherwise, results may be misleading [28]. We should caution that it is difficult to differentiate the ranking of scientists with small differences in their indicators, and this applies to both single and composite indicators.

Correction for self-citation and fractionalization of citation counts could also be considered [1], although we have not done so here. We have nevertheless explored the extent of the challenge offered by self-citations. Within a reasonable range, self-citation is not inappropriate and may be even considered necessary for transparent attribution to prior work. However, there are exceptions of extreme self-citation rates, and perhaps these should be “penalized” in the ranking. For example, “penalizing” scientists with >30% self-citation rate may affect a substantial number of scientists. Our data suggest that such a rule for selecting highly self-cited authors would have a negligible imprint on the scientists ranked at the top, based on the composite score.

New indicators are continuously proposed, and there is the challenge of how to incorporate them in bibliometric assessments of scientists, institutions, and journals [30]. With a rapid proliferation of citation metrics, there are additional indicators besides NC and H focusing on total citation impact (e.g., the Egghe g index [31]), but NC and H are still more widely used. Moreover, there are other indicators besides Hm that apply fractionalization based on the number of authors [32,33]. We used Hm, which is an index that has been widely cited since presented in 2008 (over 230 citations for the journal article and the respective arXiv article), and because some others (e.g., the h_I_ index [32]) have the undesirable paradox that they can even decrease over time (e.g., if multi-authored papers in a scientist’s curriculum vitae become more cited than her papers with few authors). Similarly, various other indicators may try to capture the citation impact of papers at specific author positions; e.g., one can calculate an H index limited to papers as single and/or first and/or last author. However, such author-position-specific H indicators may not be as informative as author-specific-position citation counts, because the number of papers for which a scientist has a specific position is limited; thus, these H indicators will mostly be very small. An H = 2 for papers as single author may refer to two papers with two citations each or two papers with 3,000 and 2,000 citations.

In several scientific fields, it has been shown that, over a researcher’s career, one moves from doing mostly implementation of research (first author) to advising, designing, and financially supporting research (last author) [34]. Moreover, authorship order reflects publication strategies of investigators [35,36]. However, attributing credit fairly according to author position may stumble upon the use of author-listing practices that do not use the first and last author positions based on maximal contributions but based on alphabetic considerations. Alphabetic listing of authors has been studied in a number of empirical evaluations, and it has been previously noted that some fields, such as economics and physics, may be particularly affected [37–39]. Our evaluation is based on a very large number of articles across all scientific fields. We show that alphabetic authorship does occur, but its impact would be tiny in most scientific fields in the life, biological, and medical sciences. A larger prevalence is seen in social sciences, humanities, physics, and mathematics. Overall, for most sub-disciplines, consideration of single, first, and last authorship offers meaningful information. However, as previous studies have shown, physics and social sciences are heterogeneous in their citation practices and in the extent to which they use alphabetic ordering. Therefore, some sub-disciplines within these fields may use alphabetic ordering far more frequently. In these sub-disciplines, use of indices based on first and last author position would be problematic. Differences between sub-disciplines may also affect the correlation pattern between different indicators. It has also been shown that in some disciplines, such as economics, first authors whose names start from ABC get more cited than those whose names start from XYZ [40]. However, the overall distortion due to this phenomenon seems very small in absolute magnitude.

The 12 key fields that we addressed here have generally good coverage at Scopus (social sciences are the one possible exception), although fields such as computer science and engineering may be at a relative disadvantage, since they have fewer citing references listed per paper. One advantage of the composite indicator is that it may help dampen differences between fields, because one type of indicator gives an advantage to some fields, but another type of indicator may counter that advantage. For example, total citations give an advantage to disciplines like empirical work in particle physics that have many multi-authored papers, but Hm dissipates this advantage, and author-order indicators even penalize this discipline. However, comparisons between fields should remain cautious. It is not even known what the ideal proportional representation of scientists from different fields among those claimed to be top scientists should be.

One other caveat is that we did not consider the age of investigators, since birth year or year of obtaining graduate degrees is generally not available. Very young investigators may have few papers and thus would be at a disadvantage to compete in these indicators against investigators with a long-standing publication record. However, in practice, it is unlikely that one would ever like to compare a first-year postdoctoral fellow against a senior professor for any reason. If insights are needed for the impact of recently published work only, one can limit the evaluation of citation indicators to the impact of papers published in the last few years. It is also well-known that for young scientists who have just published their first few papers, accrued citations don’t give a picture of their full potential [3]. Other approaches are needed to understand the potential of very young scientists [41].

We should also acknowledge that single citation indicators may have the advantage of being easier to understand and more transparent than composite indicators. For example, it is relatively straightforward to see whether a single indicator such as total citation count is very high because of a single paper with extreme citations. Conversely, a composite indicator requires more complex calculations, and the impact of single extremely-cited publications may be more difficult to discern. In some cases, the distinction between composite and non-composite indicators is not straightforward. However, a composite indicator may be more informative than any of its constituents if it captures the strengths of these constituents and does not compound their deficiencies. This may be the reason why it can also outperform single indicators in the ability to identify widely acknowledged major researchers.

Acknowledging these caveats, we argue that instead of using isolated single indicators, insights can be improved if indicators are generated systematically for the entire scientific community, and multiple such indicators are examined in parallel and in composite scores that integrate volume of citations, co-authorship, and author order. All indicators need to be used with knowledge of both their strengths and weaknesses, since none of them is perfect.

## Methods

For all citation analyses, we used the Scopus database. An XML version of the complete Scopus database was obtained in September 2014, and these data were parsed and loaded into an SQL database that was used to construct a variety of indicators at the author level. Paper-level data are available since 1900. We have considered all Scopus-indexed papers, regardless of whether they are primary research, reviews, or editorials and comments. For the 2013 citing year, 95.1% of documents cited were articles, conference papers, or reviews, and these accounted for 96.5% of the citations. Thus, so-called non-citable documents play a negligible role.

Scopus author IDs were used for all author-based analyses. We limited the analysis to citations received by scientists from papers published in 2013. A total of 10,730,738 author IDs (scientists) received at least one citation during 2013. For detailed analysis, we considered all scientists who were within the top 30,000 (roughly top 0.28%) in any of the six key indicators of interest: total number of citations received in 2013 (NC), total number of citations received in 2013 to papers for which the scientist is single author (NS), total number of citations received in 2013 to papers for which the scientist in single or first author (NSF), total number of citations received in 2013 to papers for which the scientist is single, first, or last author (NSFL), Hirsch index for the citations received in 2013 (H), and Schreiber co-authorship adjusted index for the citations received in 2013 (Hm). Hm is computed similarly to the H index after the citations of each paper have been divided by the number of authors. We also recorded the number of papers by each scientist published up to and including 2013 (NP), and the respective number of citations per paper in 2013 (CPP).

A total of 84,565 potentially eligible unique author identifiers were thus compiled that scored among the top 30,000 of each of the six key indicators. Of the 10,000 with the largest numbers of publications, we excluded 278 upon scrutiny, since they seemed to reflect cases of polysemy. Some scientists in the list had multiple author identifiers. After merging, a total of 84,116 scientists remained.

For analysis, each of the six indicators listed above was log-transformed, after inspection of the distribution of values. Several evaluations [42–44] have shown that the distributions of asymptotic number of citations to papers published in a single journal, by researchers in a single academic department, or by the same researcher all follow a discrete lognormal transformation. This offers some further justification for our use of log-transformed indicators.

Correlation analysis between the log-transformed indicators uses Pearson correlation coefficients. Analyses were performed for all scientists regardless of field and separately for each of 12 scientific fields (physics, mathematics, computer science, chemistry, earth sciences, engineering, biology/biotechnology, infectious disease, medicine, brain research, health sciences, social sciences). No analysis of indicators was performed for the humanities, since there are very few such papers in Scopus and they receive very few citations. Many scientists publish in multiple scientific fields; however, most of them have a heavy concentration in only one field. Therefore, each of the 84,116 scientists was assigned to only one field based on where he/she had the largest share of his/her published work indexed in Scopus. The median (interquartile range) for the percentage of this largest share was 76.4% (58.2% to 89.5%). The basis for assignment is the UCSD journal classification system [20]. Briefly, 554 disciplines from the UCSD map of science were aggregated into 13 high-level fields based on natural visual groupings within the map. Later, the biology and biotechnology fields were combined, and physics was split into physics and math for this study, since these two fields have widely differing authoring and citing cultures. Humanities is not represented in this analysis of top researchers.

The composite indicator is the sum of the standardized six log-transformed citation indicators (NC, H, Hm, NS, NSF, NSFL). For each standardization, a value of 1 is given to the scientist with the highest raw value for the respective indicator. Log-transformations ensure that there are no major outlier values. For parsimony, we applied equal weights to all six log-transformed indicators included in the composite. However, if, for whatever reason, one or more of these indicators are considered more essential in a particular field, one can weigh them more compared with the others.

Scientists with values for each the six metrics just below the top 30,000 that we selected may still outperform some of the selected 84,116 scientists in their composite score. We estimated what the composite score of a scientist would have been if he/she had barely missed the threshold in all six metrics. Such a scientist would still not be able to outperform the top 14,150 scientists that we have ranked according to the composite score. Therefore, the list of the top 14,150 scientists is all-inclusive, and we have not missed any scientists with higher composite scores (with the very rare exception of scientists with missed split ID records), while at lower ranks there could be additional scientists with such high composites who are not among the 84,116. We used the all-inclusive top 14,150 ranks of the composite score (and, for comparison, also of other metrics) to assess whether they capture in their lists the 47 Nobel laureates in physics, chemistry, physiology/medicine, and economics awarded in 2011–2015. While we used Nobel laureates as a measure of “ground truth” for evaluation of the different measures, there is really no perfect, gold standard on who the best scientists are, and there are limitations about any “ground truth” standard [45].

All presented indicators include self-citations. Results on the percentage of self-citations across all 84,116 scientists pertain to self-citations by the same author rather than self-citations by all authors involved in each paper.

Alphabetic order of authors is expected simply by chance in 1:k! for papers with k authors; e.g., for papers with three authors, the chances of alphabetic order of the authors simply by chance are 1/(3x2x1) = 1/6 = 16.67%.

## Supporting Information

S1 Data Indicator values for all 84,116 scientists represented in the study. (XLSX)

S1 Fig Bubble scatter plots showing the correlation between various log-adjusted citation indices among themselves. (TIF)

S1 Table Proportion of scientists from each scientific disciplines represented among those with top composite scores. Abbreviations of disciplines as in Fig 1.
(DOCX)

S2 Table Top 1,000 scientists ranked according to composite score. (DOCX)

## Abbreviations

CPP: citations per paper
H: Hirsch H index
Hm: Schreiber Hm index
NC: total citations
NP: number of papers
NS: total citations to papers for which the scientist is single author
NSF: total citations to papers for which the scientist is single or first author
NSFL: total citations to papers for which the scientist is single, first, or last author
