# Correction: Multiple Citation Indicators and Their Composite across Scientific Disciplines

**DOI:** 10.1371/journal.pbio.1002548

**Published:** 2016-08-22

**Authors:** John P.A. Ioannidis, Richard Klavans, Kevin W. Boyack

The authors would like to clarify that the correlations in Fig 1 are based on the log-transformed values for NC, H, Hm, S, SF, and SFL, using ln(val+1)/ln(max(val)+1). Np and Cpp were not log-transformed.

**Fig 1:**
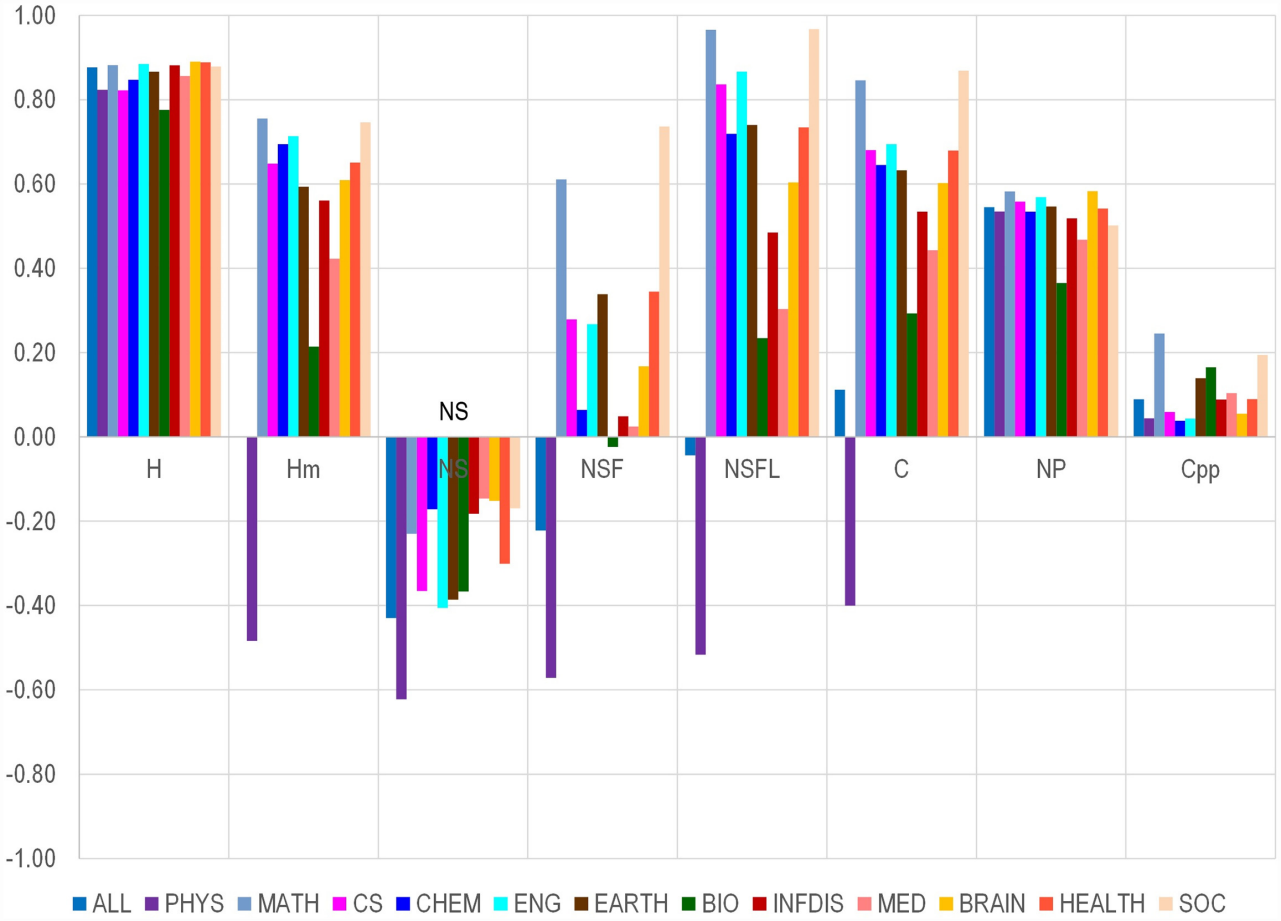
Correlation between number of citations and various citation indicators and other metrics in each of 12 different scientific fields. Abbreviations: PHYS, physics; MATH, mathematics; CS, computer science; CHEM, chemistry; ENG, engineering; EARTH, earth sciences; BIO, biology/biotechnology. INFDIS, infectious disease; MED, medicine; BRAIN, brain research; HEALTH, health sciences; SOC, social sciences. No data are shown on humanities, for which there are too few papers and too few citations in Scopus to allow meaningful analysis.
