# Supplementary figures and images for: Multiple Citation Indicators and Their Composite across Scientific Disciplines
Source: PLoS Biol. 2016 Jul 1;14(7):e1002501. doi: 10.1371/journal.pbio.1002501 (PMC4930269; doi:10.1371/journal.pbio.1002501)

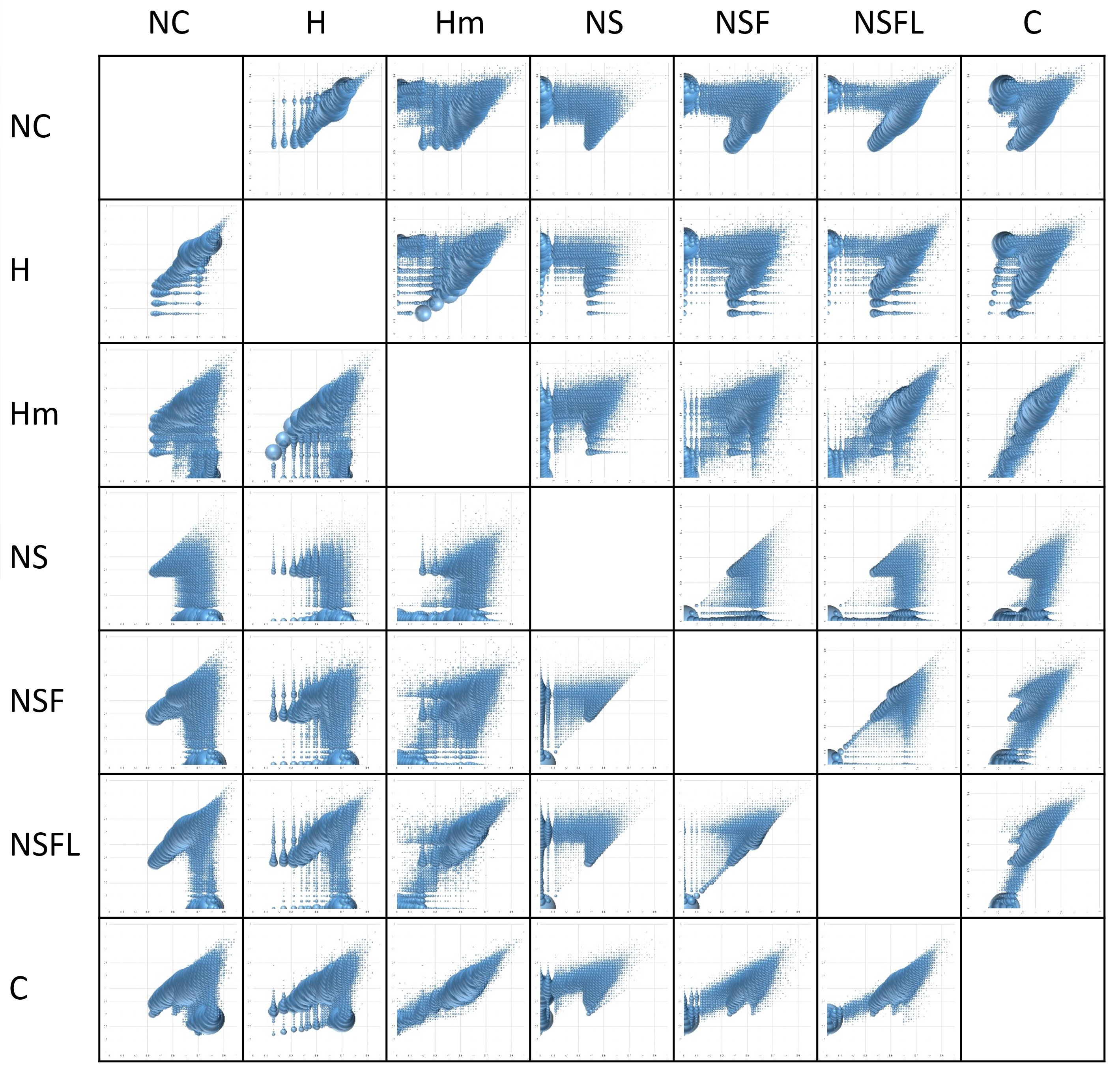

Supplement: S1 Fig — (TIF) [file pbio.1002501.s002.tif]
