# Supplementary material for: Multiple Citation Indicators and Their Composite across Scientific Disciplines
Source: PLoS Biol. 2016 Jul 1;14(7):e1002501. doi: 10.1371/journal.pbio.1002501 (PMC4930269; doi:10.1371/journal.pbio.1002501)
Supplement: S1 Table — Abbreviations of disciplines as in Fig 1. (DOCX) [file pbio.1002501.s003.docx]

|  | top1000 | top3000 | top10000 | top20000 | top30000 | All 84116 |
| --- | --- | --- | --- | --- | --- | --- |
| PHYS | 5.1% | 4.7% | 5.2% | 5.5% | 5.9% | 16.5% |
| MATH | 1.1% | 0.9% | 0.7% | 0.8% | 0.9% | 1.3% |
| CS | 3.8% | 4.0% | 4.5% | 4.7% | 4.9% | 5.3% |
| CHEM | 13.9% | 13.2% | 11.1% | 10.6% | 10.6% | 10.4% |
| ENG | 3.8% | 4.0% | 4.1% | 4.6% | 5.0% | 5.5% |
| EARTH | 1.5% | 2.3% | 3.6% | 4.2% | 4.4% | 3.6% |
| BIO | 10.0% | 9.8% | 9.9% | 10.3% | 10.4% | 9.1% |
| INFDIS | 3.1% | 3.6% | 4.2% | 4.2% | 4.2% | 3.4% |
| MED | 32.4% | 32.4% | 32.8% | 31.9% | 30.8% | 25.1% |
| BRAIN | 15.4% | 14.3% | 12.7% | 11.8% | 11.2% | 7.8% |
| HEALTH | 4.2% | 5.2% | 5.6% | 5.7% | 5.7% | 4.6% |
| SOC | 5.7% | 5.6% | 5.5% | 5.8% | 6.0% | 7.4% |
