# Supplementary material for: Multiple Citation Indicators and Their Composite across Scientific Disciplines
Source: PLoS Biol. 2016 Jul 1;14(7):e1002501. doi: 10.1371/journal.pbio.1002501 (PMC4930269; doi:10.1371/journal.pbio.1002501)
Supplement: S2 Table — (DOCX) [file pbio.1002501.s004.docx]

| **Author** | **Field** | **NC** | **H** | **Hm** | **NS** | **NSF** | **NSFL** |
| --- | --- | --- | --- | --- | --- | --- | --- |
| Gratzel M. | CHEM | 19245 | 61 | 30.285 | 2206 | 2223 | 15103 |
| Bartel D.P. | BIO | 9818 | 42 | 17.375 | 2952 | 3041 | 7661 |
| Newman M.E.J. | MATH | 5938 | 33 | 24.950 | 2941 | 3971 | 5473 |
| Kessler R.C. | HEALTH | 11707 | 49 | 23.990 | 297 | 5815 | 8802 |
| Wang Z.L. | PHYS | 9407 | 43 | 26.597 | 1063 | 1581 | 7282 |
| Becke A.D. | CHEM | 8390 | 17 | 13.000 | 7823 | 8091 | 8237 |
| Semenza G.L. | MED | 5777 | 38 | 28.955 | 1842 | 2166 | 4427 |
| Geim A.K. | PHYS | 16164 | 39 | 14.391 | 975 | 3788 | 8400 |
| Friston K.J. | BRAIN | 8511 | 41 | 24.767 | 741 | 2764 | 5885 |
| Whitesides G.M. | CHEM | 11300 | 47 | 24.900 | 515 | 1336 | 9804 |
| McEwen B.S. | BRAIN | 6960 | 40 | 27.844 | 1202 | 1993 | 4244 |
| Zadeh L.A. | CS | 4793 | 21 | 21.000 | 4756 | 4756 | 4776 |
| Altman D.G. | MED | 16490 | 57 | 28.302 | 222 | 993 | 8679 |
| Perdew J.P. | CHEM | 12145 | 23 | 12.611 | 941 | 11239 | 11695 |
| Halliwell B. | MED | 4881 | 35 | 26.149 | 1460 | 2790 | 4096 |
| Donoho D.L. | CS | 5866 | 30 | 19.533 | 2418 | 3751 | 4446 |
| Holick M.F. | MED | 5127 | 32 | 21.077 | 1875 | 2763 | 4242 |
| Akira S. | INFDIS | 15424 | 53 | 24.674 | 169 | 1400 | 7466 |
| Karin M. | MED | 8354 | 44 | 25.728 | 407 | 1260 | 6683 |
| Langer R. | CHEM | 11892 | 48 | 25.066 | 286 | 1110 | 6346 |
| Jain R.K. | MED | 6912 | 38 | 21.947 | 879 | 1422 | 5577 |
| Selkoe D.J. | BRAIN | 5910 | 34 | 22.828 | 1257 | 1415 | 4647 |
| Alivisatos A.P. | CHEM | 7297 | 41 | 21.462 | 770 | 1112 | 6293 |
| Ridker P.M. | MED | 9143 | 46 | 19.959 | 333 | 2715 | 4576 |
| Novoselov K.S. | PHYS | 15628 | 39 | 14.955 | 107 | 6083 | 10260 |
| Kamat P.V. | CHEM | 4862 | 36 | 24.014 | 1016 | 1299 | 4540 |
| Corma A. | CHEM | 7157 | 33 | 19.316 | 543 | 3212 | 5030 |
| Hu F.B. | MED | 10368 | 45 | 22.670 | 293 | 1320 | 5154 |
| Folkman J. | MED | 4708 | 31 | 20.519 | 1509 | 2010 | 3597 |
| Willett W.C. | MED | 14265 | 47 | 24.051 | 205 | 935 | 5047 |
| Grimme S. | CHEM | 4388 | 27 | 18.754 | 1848 | 2491 | 3526 |
| Ferrara N. | MED | 5660 | 35 | 21.858 | 701 | 2040 | 3478 |
| Lander E.S. | MED | 15766 | 68 | 17.896 | 126 | 1318 | 4712 |
| Barnes P.J. | MED | 6393 | 30 | 19.901 | 1108 | 1737 | 3746 |
| Barabasi A.-L. | MATH | 7354 | 35 | 19.335 | 325 | 2594 | 6002 |
| Bandura A. | SOC | 3337 | 24 | 19.348 | 2357 | 2904 | 3100 |
| Libby P. | MED | 6590 | 34 | 19.975 | 772 | 1728 | 3475 |
| Witten E. | PHYS | 2790 | 27 | 23.583 | 1739 | 1755 | 2787 |
| Nei M. | BIO | 14268 | 24 | 15.500 | 719 | 1763 | 5633 |
| Smith S.M. | BRAIN | 6767 | 40 | 13.992 | 531 | 2443 | 4659 |
| Xia Y. | CHEM | 11432 | 49 | 27.849 | 20 | 1779 | 10147 |
| Clevers H. | MED | 6571 | 41 | 18.911 | 538 | 823 | 4669 |
| Guyatt G.H. | MED | 8757 | 40 | 17.871 | 180 | 2358 | 4606 |
| Croce C.M. | MED | 11138 | 51 | 19.756 | 294 | 366 | 6231 |
| Medzhitov R. | INFDIS | 4314 | 37 | 20.752 | 562 | 1310 | 3614 |
| Tilman D. | BIO | 4878 | 37 | 20.609 | 475 | 1772 | 2839 |
| Hotamisligil G.S. | MED | 4620 | 33 | 17.884 | 794 | 1583 | 3614 |
| Kroemer G. | MED | 10057 | 46 | 22.914 | 62 | 1247 | 7801 |
| Massague J. | MED | 4679 | 36 | 19.121 | 680 | 1107 | 3973 |
| Candes E.J. | CS | 5570 | 25 | 15.367 | 383 | 5027 | 5144 |
| Aggarwal B.B. | MED | 5990 | 35 | 21.092 | 273 | 1405 | 5163 |
| Ioannidis J.P.A. | MED | 6901 | 31 | 18.367 | 669 | 1527 | 2973 |
| Mizushima N. | MED | 6342 | 41 | 16.867 | 350 | 1998 | 3042 |
| Mattson M.P. | BRAIN | 5401 | 29 | 19.847 | 616 | 1394 | 3712 |
| Sheldrick G.M. | CHEM | 7329 | 9 | 6.946 | 6847 | 6897 | 7109 |
| Demirbas A. | ENG | 2066 | 24 | 23.200 | 1766 | 1924 | 2000 |
| Iijima S. | CHEM | 5430 | 21 | 10.807 | 2063 | 2532 | 4736 |
| Dai H. | CHEM | 8996 | 52 | 20.906 | 159 | 364 | 7687 |
| Pendry J.B. | PHYS | 4548 | 28 | 15.209 | 871 | 2371 | 3196 |
| Grundy S.M. | MED | 6785 | 35 | 14.766 | 438 | 2302 | 2895 |
| Carmeliet P. | MED | 5401 | 32 | 14.975 | 610 | 1878 | 3489 |
| Petersen R.C. | BRAIN | 8043 | 39 | 14.347 | 432 | 1556 | 2788 |
| Barker D.J.P. | MED | 3722 | 29 | 18.423 | 817 | 1731 | 2653 |
| Esteller M. | MED | 4574 | 31 | 16.909 | 765 | 1259 | 3283 |
| Mantovani A. | MED | 7159 | 35 | 17.945 | 160 | 1942 | 4628 |
| Diener E. | SOC | 3908 | 28 | 17.726 | 472 | 2446 | 3398 |
| Giovannucci E. | MED | 7176 | 34 | 19.040 | 408 | 1166 | 2405 |
| Lowe D.G. | CS | 4165 | 15 | 9.811 | 3573 | 3574 | 4062 |
| Chou K.-C. | BIO | 3429 | 25 | 19.886 | 715 | 1559 | 3299 |
| Forrest S.R. | PHYS | 5149 | 35 | 19.971 | 476 | 616 | 4092 |
| Dinarello C.A. | MED | 4205 | 25 | 18.337 | 1068 | 1346 | 2227 |
| Weissleder R. | MED | 6921 | 37 | 18.727 | 259 | 939 | 3548 |
| Matyjaszewski K. | CHEM | 6140 | 31 | 18.529 | 235 | 1166 | 5286 |
| Kannel W.B. | MED | 5832 | 36 | 17.828 | 243 | 1448 | 3400 |
| Parkin D.M. | MED | 5541 | 23 | 14.554 | 473 | 2488 | 4566 |
| Trost B.M. | CHEM | 3685 | 20 | 15.833 | 533 | 3601 | 3659 |
| Heeger A.J. | CHEM | 8145 | 39 | 18.901 | 260 | 357 | 5664 |
| Lip G.Y.H. | MED | 9553 | 40 | 15.792 | 126 | 1279 | 4270 |
| El-Sayed M.A. | CHEM | 6538 | 39 | 21.218 | 273 | 278 | 6278 |
| Yusuf S. | MED | 9650 | 50 | 14.964 | 139 | 717 | 4721 |
| Braunwald E. | MED | 7852 | 43 | 15.444 | 286 | 576 | 4277 |
| Ford E.S. | MED | 6990 | 30 | 17.795 | 321 | 1813 | 1962 |
| Ostrom E. | SOC | 2591 | 23 | 16.632 | 1535 | 1731 | 2126 |
| DeFronzo R.A. | MED | 4530 | 30 | 16.469 | 442 | 1544 | 2940 |
| Gage F.H. | BRAIN | 7224 | 42 | 20.759 | 225 | 329 | 4846 |
| El-Serag H.B. | MED | 3402 | 27 | 16.855 | 574 | 1765 | 2809 |
| Nestler E.J. | BRAIN | 4864 | 35 | 18.908 | 319 | 754 | 3622 |
| Egger M. | MED | 8007 | 36 | 15.998 | 105 | 2209 | 3504 |
| Xu Z. | CS | 1973 | 25 | 20.917 | 980 | 1686 | 1808 |
| Torchilin V.P. | CHEM | 3059 | 25 | 17.037 | 1100 | 1330 | 2283 |
| Robbins T.W. | BRAIN | 6991 | 35 | 21.943 | 178 | 583 | 4085 |
| Wang J. | CHEM | 3487 | 23 | 17.442 | 618 | 1722 | 2893 |
| Buzsaki G. | BRAIN | 3346 | 29 | 20.999 | 405 | 1053 | 2847 |
| Mann M. | BIO | 10073 | 45 | 22.871 | 69 | 310 | 6702 |
| Baddeley A.D. | BRAIN | 2471 | 22 | 18.326 | 1021 | 1716 | 2174 |
| Yang Z. | BIO | 2519 | 24 | 17.435 | 908 | 1621 | 2319 |
| Posada D. | BIO | 4272 | 19 | 11.978 | 1149 | 2422 | 3094 |
| LeDoux J.E. | BRAIN | 3171 | 29 | 18.802 | 697 | 822 | 2543 |
| Zhu J.-K. | BIO | 3923 | 33 | 18.983 | 526 | 571 | 2960 |
| Ryan R.M. | SOC | 4679 | 33 | 19.743 | 105 | 1808 | 3740 |
| Tarascon J.-M. | ENG | 7646 | 35 | 16.903 | 83 | 1230 | 5815 |
| Reiter R.J. | HEALTH | 4939 | 27 | 17.993 | 360 | 1136 | 2887 |
| Serhan C.N. | MED | 4125 | 33 | 17.468 | 267 | 1268 | 2864 |
| Morris J.C. | BRAIN | 5965 | 35 | 14.275 | 451 | 945 | 2212 |
| Levey A.S. | MED | 8285 | 38 | 12.705 | 39 | 3804 | 5653 |
| Gross J.J. | BRAIN | 3109 | 28 | 17.618 | 553 | 1037 | 2662 |
| Krebs F.C. | CHEM | 3415 | 28 | 16.012 | 490 | 1136 | 3174 |
| Jain A.K. | CS | 3478 | 26 | 17.933 | 330 | 1485 | 3120 |
| De Clercq E. | CHEM | 4762 | 23 | 16.918 | 728 | 951 | 2366 |
| Kresse G. | PHYS | 10630 | 20 | 11.969 | 30 | 9433 | 9966 |
| Robertson J. | PHYS | 4271 | 23 | 15.556 | 732 | 994 | 3083 |
| Kendler K.S. | BRAIN | 4847 | 28 | 15.851 | 193 | 1987 | 3440 |
| Yager R.R. | CS | 1912 | 21 | 18.667 | 1255 | 1476 | 1853 |
| Green M.A. | PHYS | 3040 | 26 | 17.010 | 450 | 1577 | 2550 |
| Yang P. | PHYS | 7957 | 46 | 20.730 | 39 | 580 | 6242 |
| Thompson C.B. | MED | 7239 | 41 | 18.772 | 192 | 291 | 4300 |
| Koonin E.V. | BIO | 5965 | 32 | 17.730 | 263 | 711 | 2959 |
| Sakaguchi S. | INFDIS | 3706 | 31 | 14.969 | 348 | 1298 | 3132 |
| Baumeister R.F. | SOC | 3359 | 29 | 18.325 | 214 | 1843 | 2712 |
| Reed J.C. | MED | 5859 | 31 | 17.753 | 384 | 497 | 3014 |
| Logan B.E. | BIO | 3864 | 30 | 18.998 | 230 | 961 | 3558 |
| Koob G.F. | BRAIN | 3773 | 24 | 18.109 | 400 | 1327 | 2652 |
| Rutter M. | SOC | 3910 | 25 | 16.805 | 613 | 1038 | 2295 |
| Hartwig J.F. | CHEM | 3484 | 27 | 19.933 | 440 | 589 | 3361 |
| Frith C.D. | BRAIN | 5665 | 35 | 19.812 | 161 | 638 | 3188 |
| Beck A.T. | HEALTH | 4075 | 26 | 13.770 | 178 | 3078 | 3771 |
| Ferey G. | CHEM | 4229 | 31 | 12.897 | 560 | 987 | 2737 |
| Lovley D.R. | BIO | 3451 | 27 | 16.698 | 390 | 1024 | 3140 |
| Vincent J.-L. | MED | 5819 | 30 | 13.711 | 173 | 1553 | 3841 |
| Hardie D.G. | MED | 2943 | 28 | 16.821 | 577 | 1025 | 2132 |
| Lieber C.M. | PHYS | 6852 | 44 | 23.822 | 118 | 124 | 6438 |
| Sheldon R.A. | CHEM | 2429 | 24 | 17.736 | 714 | 1146 | 2183 |
| Weinberg R.A. | MED | 9263 | 36 | 17.272 | 161 | 176 | 8615 |
| Tsien R.Y. | MED | 4294 | 33 | 17.137 | 390 | 448 | 3332 |
| Baselga J. | MED | 6609 | 40 | 14.885 | 118 | 1207 | 2666 |
| Kahneman D. | SOC | 3528 | 25 | 16.017 | 302 | 1476 | 3390 |
| Ajayan P.M. | CHEM | 5456 | 35 | 16.834 | 187 | 618 | 3701 |
| Marmot M. | HEALTH | 4943 | 29 | 15.592 | 311 | 964 | 2744 |
| Hardy J. | BRAIN | 6558 | 36 | 13.482 | 220 | 1340 | 1906 |
| Lal R. | ENG | 2557 | 19 | 16.629 | 1131 | 1279 | 1993 |
| Klionsky D.J. | MED | 4089 | 30 | 15.987 | 203 | 1102 | 3776 |
| Burnstock G. | MED | 2977 | 22 | 16.684 | 750 | 1081 | 2247 |
| Dubey J.P. | BIO | 3321 | 23 | 15.907 | 434 | 1608 | 2456 |
| Maron B.J. | MED | 3782 | 29 | 13.269 | 330 | 1710 | 2610 |
| Spiegelman B.M. | MED | 6914 | 47 | 18.879 | 80 | 294 | 5437 |
| Ferrari A.C. | PHYS | 4748 | 29 | 10.729 | 380 | 1653 | 3191 |
| Raichle M.E. | BRAIN | 5195 | 33 | 15.845 | 132 | 1072 | 3841 |
| Yaghi O.M. | CHEM | 8325 | 46 | 18.996 | 18 | 797 | 7819 |
| Springel V. | PHYS | 3948 | 29 | 14.723 | 385 | 1295 | 2081 |
| Jorgensen W.L. | CHEM | 3933 | 19 | 14.867 | 244 | 2740 | 3804 |
| Thomson J.A. | MED | 4633 | 32 | 11.153 | 705 | 781 | 2559 |
| Ingber D.E. | INFDIS | 3475 | 29 | 17.527 | 480 | 587 | 2521 |
| Nowak M.A. | MATH | 3676 | 29 | 15.951 | 279 | 1243 | 2503 |
| Hannon G.J. | MED | 7536 | 45 | 18.665 | 157 | 245 | 3051 |
| Rosenberg S.A. | MED | 5472 | 34 | 14.896 | 161 | 894 | 3560 |
| Hanahan D. | MED | 5724 | 26 | 12.722 | 71 | 3769 | 5011 |
| Calder P.C. | HEALTH | 2487 | 22 | 17.241 | 786 | 1106 | 1936 |
| Moffitt T.E. | SOC | 4878 | 31 | 16.084 | 353 | 805 | 1811 |
| Cantley L.C. | MED | 6609 | 37 | 15.389 | 267 | 383 | 2926 |
| Goodenough J.B. | PHYS | 4021 | 25 | 16.189 | 297 | 977 | 3326 |
| Ajzen I. | SOC | 2594 | 14 | 10.667 | 2067 | 2421 | 2538 |
| Rothwell P.M. | MED | 3311 | 29 | 15.407 | 259 | 1393 | 2603 |
| Levine B. | MED | 4962 | 37 | 15.428 | 90 | 1235 | 3561 |
| Bird A. | MED | 2981 | 28 | 14.379 | 647 | 743 | 2627 |
| Mirkin C.A. | CHEM | 7493 | 41 | 21.560 | 41 | 365 | 5972 |
| Collins F.S. | MED | 10605 | 43 | 13.397 | 161 | 583 | 1888 |
| Buckner R.L. | BRAIN | 4506 | 35 | 15.157 | 111 | 1326 | 3118 |
| Gordon S. | MED | 3721 | 25 | 14.649 | 445 | 1017 | 2497 |
| Levy D. | MED | 8522 | 43 | 17.476 | 41 | 785 | 3446 |
| Popkin B.M. | HEALTH | 3091 | 24 | 18.909 | 370 | 760 | 2601 |
| Green D.R. | MED | 4847 | 34 | 17.849 | 106 | 892 | 2887 |
| Caplan A.I. | MED | 2866 | 28 | 15.482 | 546 | 883 | 1885 |
| Dehaene S. | BRAIN | 3340 | 30 | 17.904 | 153 | 1300 | 2489 |
| Vitousek P.M. | BIO | 3165 | 31 | 15.458 | 197 | 1464 | 2419 |
| Hunter D.J. | MED | 9108 | 38 | 14.790 | 118 | 757 | 2060 |
| Crabtree R.H. | CHEM | 2732 | 25 | 17.944 | 575 | 706 | 1977 |
| Rubin D.B. | SOC | 2623 | 19 | 15.131 | 855 | 1002 | 2581 |
| Volkow N.D. | BRAIN | 4815 | 31 | 17.700 | 42 | 2144 | 3664 |
| Li C.-J. | CHEM | 2566 | 24 | 16.702 | 566 | 769 | 2480 |
| Ren W. | CS | 2238 | 23 | 17.650 | 442 | 1486 | 1717 |
| Mosmann T.R. | INFDIS | 3213 | 15 | 7.551 | 2110 | 2534 | 2844 |
| Hallett M. | BRAIN | 5465 | 30 | 15.744 | 301 | 397 | 3132 |
| Sudhof T.C. | BRAIN | 4304 | 28 | 16.767 | 327 | 522 | 2663 |
| Baron-Cohen S. | BRAIN | 3728 | 25 | 15.833 | 206 | 1602 | 2356 |
| Guarente L. | MED | 3678 | 37 | 18.220 | 243 | 353 | 2736 |
| Jemal A. | MED | 14391 | 35 | 11.036 | 2 | 8378 | 12865 |
| Davidson R.J. | BRAIN | 3181 | 27 | 16.783 | 298 | 839 | 2480 |
| Mancia G. | MED | 6384 | 32 | 12.967 | 69 | 1860 | 3528 |
| Olson E.N. | MED | 6422 | 39 | 19.902 | 101 | 214 | 4481 |
| Ackermann L. | CHEM | 2031 | 23 | 15.408 | 459 | 1691 | 1982 |
| Uversky V.N. | BIO | 2909 | 27 | 15.751 | 413 | 1067 | 1608 |
| Mardis E.R. | BIO | 9882 | 46 | 9.912 | 399 | 572 | 958 |
| Cole T.J. | MED | 3906 | 24 | 14.471 | 215 | 1675 | 2391 |
| Tversky A. | SOC | 2789 | 22 | 13.833 | 274 | 2053 | 2767 |
| Mesulam M.-M. | BRAIN | 3015 | 24 | 16.041 | 489 | 897 | 1815 |
| Dobson C.M. | BIO | 4279 | 28 | 15.024 | 419 | 522 | 2251 |
| Colditz G.A. | MED | 8408 | 37 | 19.550 | 54 | 603 | 2386 |
| Turner R.C. | MED | 4204 | 18 | 7.559 | 1129 | 1673 | 3512 |
| Spek A.L. | CHEM | 4122 | 14 | 7.924 | 2009 | 2012 | 2418 |
| Woolf C.J. | BRAIN | 3116 | 29 | 15.324 | 302 | 798 | 2336 |
| Ambros V. | BIO | 2637 | 21 | 12.959 | 797 | 986 | 2418 |
| Weinberg S. | PHYS | 1436 | 19 | 16.667 | 1215 | 1215 | 1434 |
| Felson D.T. | MED | 4828 | 29 | 14.335 | 163 | 1149 | 2338 |
| Schultz W. | BRAIN | 1934 | 22 | 15.683 | 661 | 1151 | 1827 |
| Kanis J.A. | MED | 4377 | 33 | 13.435 | 99 | 1639 | 2654 |
| Gupta V.K. | CHEM | 4502 | 39 | 22.760 | 7 | 3077 | 3456 |
| Cummings J.L. | BRAIN | 4942 | 28 | 13.197 | 353 | 884 | 1650 |
| Steinman R.M. | INFDIS | 3691 | 26 | 14.941 | 245 | 918 | 2576 |
| Vanderbilt D. | PHYS | 2459 | 21 | 12.350 | 923 | 1032 | 2129 |
| White N.J. | INFDIS | 4457 | 24 | 14.740 | 453 | 585 | 2106 |
| Jones P.A. | MED | 3266 | 25 | 15.237 | 215 | 1050 | 2896 |
| Greenland S. | HEALTH | 2096 | 22 | 16.230 | 562 | 1080 | 1674 |
| Drucker D.J. | MED | 3072 | 26 | 14.625 | 360 | 904 | 2036 |
| Davis M.E. | CHEM | 2736 | 23 | 13.726 | 402 | 1080 | 2503 |
| Hancock R.E.W. | INFDIS | 3866 | 26 | 17.391 | 169 | 765 | 2633 |
| Berridge K.C. | BRAIN | 2110 | 25 | 17.617 | 386 | 811 | 1933 |
| Bentler P.M. | SOC | 4527 | 12 | 9.833 | 902 | 1419 | 4470 |
| Laemmli U.K. | MED | 3930 | 8 | 5.525 | 3696 | 3743 | 3917 |
| Ruoff R.S. | CHEM | 11917 | 46 | 15.592 | 48 | 97 | 10111 |
| Adolphs R. | BRAIN | 2070 | 25 | 15.795 | 517 | 993 | 1489 |
| Krumholz H.M. | MED | 6470 | 35 | 14.942 | 83 | 623 | 3047 |
| Yamanaka S. | MED | 5437 | 29 | 13.914 | 222 | 328 | 4457 |
| Sandborn W.J. | MED | 5046 | 38 | 13.900 | 76 | 991 | 2643 |
| McMurray J.J.V. | MED | 8845 | 41 | 13.682 | 35 | 1210 | 2461 |
| Furstner A. | CHEM | 2273 | 21 | 15.711 | 308 | 1494 | 2226 |
| Wallace D.C. | MED | 2487 | 25 | 14.240 | 549 | 786 | 1882 |
| Ding F. | CS | 2041 | 28 | 19.143 | 181 | 1207 | 1686 |
| Prockop D.J. | MED | 3816 | 29 | 13.603 | 345 | 552 | 2290 |
| Truhlar D.G. | CHEM | 6616 | 33 | 19.703 | 50 | 269 | 6202 |
| Watson D. | HEALTH | 3217 | 23 | 13.760 | 140 | 2138 | 2786 |
| Trenberth K.E. | EARTH | 2366 | 24 | 15.772 | 370 | 1192 | 1504 |
| Meaney M.J. | BRAIN | 3550 | 29 | 16.971 | 194 | 488 | 2712 |
| Moher D. | MED | 7941 | 37 | 13.860 | 9 | 2799 | 4876 |
| Inoue A. | ENG | 3929 | 19 | 12.785 | 335 | 1096 | 3091 |
| Holst J.J. | MED | 5052 | 29 | 15.566 | 251 | 508 | 1729 |
| Posner M.I. | BRAIN | 2573 | 23 | 14.162 | 329 | 1129 | 2335 |
| Vasan R.S. | MED | 7078 | 39 | 13.964 | 74 | 574 | 2769 |
| Murray C.J.L. | HEALTH | 4778 | 29 | 12.886 | 60 | 1722 | 3860 |
| Sun S. | CHEM | 3589 | 32 | 16.201 | 88 | 855 | 3082 |
| Kappe C.O. | CHEM | 2172 | 21 | 14.617 | 598 | 903 | 2008 |
| Ashburner J. | BRAIN | 3241 | 25 | 11.838 | 356 | 1495 | 1657 |
| Tracey K.J. | MED | 3569 | 30 | 15.068 | 296 | 447 | 2236 |
| Haruta M. | CHEM | 2396 | 24 | 14.008 | 493 | 883 | 1850 |
| Supuran C.T. | CHEM | 3768 | 21 | 13.398 | 412 | 693 | 2767 |
| Breiman L. | CS | 2627 | 9 | 8.500 | 2595 | 2617 | 2625 |
| Melton III L.J. | MED | 5604 | 31 | 15.501 | 101 | 566 | 2948 |
| Steinberg L. | SOC | 2015 | 25 | 14.926 | 488 | 1003 | 1446 |
| Prusiner S.B. | INFDIS | 2908 | 22 | 12.986 | 610 | 748 | 2032 |
| Deci E.L. | SOC | 3687 | 27 | 13.919 | 89 | 1403 | 3489 |
| Sapolsky R.M. | BRAIN | 2162 | 21 | 16.511 | 437 | 984 | 1707 |
| Raoult D. | INFDIS | 6488 | 26 | 16.634 | 77 | 504 | 4483 |
| Carver C.S. | SOC | 2454 | 21 | 14.371 | 321 | 1414 | 2037 |
| Bates D.W. | HEALTH | 4339 | 30 | 15.018 | 91 | 860 | 3031 |
| Neese F. | CHEM | 2599 | 22 | 16.427 | 450 | 720 | 1693 |
| Jankovic J. | BRAIN | 3714 | 25 | 13.960 | 330 | 798 | 1732 |
| Rayner K. | BRAIN | 2139 | 19 | 14.933 | 527 | 1102 | 1922 |
| Brus L.E. | CHEM | 2623 | 29 | 14.780 | 452 | 459 | 1836 |
| Finkel T. | MED | 2994 | 27 | 12.457 | 312 | 859 | 2290 |
| Venkatesh V. | SOC | 2479 | 20 | 13.533 | 236 | 2067 | 2327 |
| Nathan C. | MED | 2282 | 22 | 15.624 | 465 | 842 | 1716 |
| Ganz T. | INFDIS | 2921 | 25 | 14.527 | 444 | 715 | 1515 |
| Beal M.F. | BRAIN | 3768 | 26 | 15.759 | 303 | 463 | 2064 |
| Tefferi A. | MED | 3604 | 23 | 13.235 | 256 | 943 | 2528 |
| Sallis J.F. | HEALTH | 5230 | 35 | 14.660 | 67 | 1193 | 1749 |
| Kehlet H. | MED | 3304 | 24 | 15.150 | 212 | 803 | 2530 |
| Sampson H.A. | MED | 3171 | 27 | 14.911 | 274 | 629 | 2068 |
| Tibshirani R. | MED | 5465 | 30 | 15.358 | 118 | 513 | 2697 |
| Hashmi A.S.K. | CHEM | 2088 | 19 | 14.513 | 359 | 1649 | 1974 |
| Eddy S.R. | BIO | 3990 | 25 | 12.900 | 539 | 566 | 1526 |
| Topol E.J. | MED | 4991 | 32 | 13.552 | 187 | 377 | 2931 |
| Ames B.N. | MED | 2850 | 24 | 15.925 | 212 | 774 | 2595 |
| Hochberg M.C. | MED | 4024 | 26 | 10.958 | 565 | 865 | 1281 |
| Hell S.W. | CS | 3009 | 25 | 13.713 | 340 | 682 | 2230 |
| Brownlee M. | MED | 2471 | 21 | 12.216 | 750 | 885 | 1744 |
| Desiraju G.R. | CHEM | 1827 | 20 | 15.571 | 730 | 813 | 1522 |
| Jaenisch R. | MED | 7748 | 45 | 17.883 | 20 | 376 | 4332 |
| Dunning Jr. T.H. | CHEM | 2978 | 15 | 8.799 | 1358 | 1368 | 2263 |
| Levine A.J. | MED | 4031 | 28 | 13.872 | 229 | 576 | 2209 |
| Lehn J.-M. | CHEM | 2397 | 20 | 15.238 | 620 | 698 | 1674 |
| Sies H. | MED | 2641 | 23 | 14.760 | 430 | 691 | 1826 |
| Holgate S.T. | MED | 3281 | 25 | 14.402 | 355 | 708 | 1563 |
| Duman R.S. | BRAIN | 3185 | 29 | 17.215 | 135 | 592 | 2449 |
| Friedman S.L. | MED | 2484 | 21 | 14.858 | 641 | 759 | 1345 |
| Kroenke K. | HEALTH | 3716 | 26 | 13.522 | 95 | 1743 | 2359 |
| Wishart D.S. | CHEM | 2540 | 28 | 13.926 | 184 | 1098 | 2142 |
| Nathan D.M. | MED | 4407 | 30 | 10.400 | 146 | 1282 | 2492 |
| Smith G.D. | MED | 8288 | 32 | 14.984 | 88 | 472 | 2175 |
| Rakic P. | BRAIN | 2196 | 23 | 16.099 | 477 | 630 | 1635 |
| Fridovich I. | BIO | 2681 | 19 | 16.667 | 462 | 469 | 2599 |
| De Gennes P.-G. | CHEM | 1495 | 18 | 14.583 | 957 | 1082 | 1401 |
| Kouzarides T. | MED | 2516 | 23 | 11.966 | 668 | 676 | 1943 |
| Hansson G.K. | MED | 2595 | 21 | 12.856 | 492 | 917 | 1927 |
| Maier J. | ENG | 3320 | 29 | 14.988 | 291 | 312 | 2673 |
| McClements D.J. | CHEM | 2779 | 21 | 16.650 | 308 | 652 | 2143 |
| Dietz W.H. | HEALTH | 4031 | 26 | 14.638 | 243 | 380 | 2938 |
| Marsh H.W. | SOC | 1968 | 21 | 14.936 | 306 | 1482 | 1715 |
| Flegal K.M. | MED | 6082 | 30 | 11.897 | 45 | 1677 | 3138 |
| Biederman J. | BRAIN | 4581 | 25 | 13.863 | 91 | 1476 | 2244 |
| Decety J. | BRAIN | 2226 | 26 | 17.804 | 165 | 927 | 1791 |
| Wallentin L. | MED | 7426 | 38 | 10.982 | 53 | 787 | 3327 |
| Hynes R.O. | MED | 2319 | 20 | 12.592 | 788 | 859 | 1497 |
| Klein R. | MED | 5757 | 33 | 14.998 | 43 | 1306 | 1858 |
| Bhushan B. | ENG | 2462 | 20 | 15.617 | 279 | 940 | 2148 |
| Bechara A. | BRAIN | 2541 | 25 | 14.270 | 182 | 1260 | 1878 |
| Fredrickson B.L. | SOC | 1775 | 22 | 12.568 | 546 | 1222 | 1539 |
| Weinberger D.R. | BRAIN | 5349 | 32 | 14.641 | 136 | 271 | 3231 |
| Goldman-Rakic P.S. | BRAIN | 2195 | 23 | 17.017 | 353 | 520 | 1938 |
| Pritchard J.K. | BIO | 4198 | 27 | 11.706 | 59 | 1842 | 3514 |
| Pedersen B.K. | HEALTH | 3090 | 25 | 15.183 | 190 | 717 | 2167 |
| Dale A.M. | BRAIN | 5407 | 33 | 13.585 | 74 | 600 | 2990 |
| Teece D.J. | SOC | 1777 | 14 | 12.583 | 888 | 1589 | 1773 |
| Engelman J.A. | MED | 4165 | 33 | 9.227 | 202 | 1120 | 1853 |
| Angell C.A. | CHEM | 1914 | 21 | 15.126 | 489 | 806 | 1616 |
| Harris A.L. | MED | 5689 | 29 | 13.827 | 233 | 271 | 2364 |
| Ernst E. | HEALTH | 3443 | 17 | 12.863 | 474 | 826 | 2177 |
| Pfaffl M.W. | INFDIS | 3349 | 13 | 5.844 | 1649 | 2299 | 2593 |
| Kudo A. | CHEM | 2712 | 25 | 17.801 | 97 | 1025 | 2201 |
| Ruoslahti E. | MED | 2783 | 26 | 14.515 | 286 | 546 | 2022 |
| Mittler R. | BIO | 2151 | 23 | 11.421 | 505 | 1030 | 1762 |
| Van Der Heijde D. | MED | 4551 | 33 | 12.390 | 119 | 751 | 1979 |
| Chrousos G.P. | MED | 4182 | 25 | 14.963 | 181 | 459 | 2551 |
| Reaven G.M. | MED | 2057 | 19 | 13.222 | 667 | 866 | 1621 |
| Wang L.V. | CS | 3480 | 26 | 16.275 | 168 | 418 | 2619 |
| Wittchen H.-U. | HEALTH | 3856 | 28 | 13.668 | 142 | 829 | 1878 |
| Rolls E.T. | BRAIN | 2362 | 21 | 14.935 | 387 | 894 | 1418 |
| Watts D.J. | MATH | 2834 | 20 | 11.867 | 165 | 1864 | 2766 |
| Zhao G. | EARTH | 3363 | 29 | 17.923 | 61 | 1263 | 1518 |
| Avouris P. | PHYS | 3009 | 28 | 12.947 | 196 | 631 | 2508 |
| Canfield D.E. | EARTH | 1823 | 25 | 16.800 | 277 | 812 | 1407 |
| Llovet J.M. | MED | 4491 | 32 | 12.021 | 30 | 2185 | 3075 |
| Bruce P.G. | ENG | 4277 | 30 | 15.635 | 40 | 925 | 3339 |
| Squire L.R. | BRAIN | 2269 | 22 | 14.625 | 299 | 845 | 1880 |
| Bisquert J. | CHEM | 2641 | 25 | 13.797 | 267 | 791 | 1714 |
| Fuster V. | MED | 5501 | 33 | 12.845 | 55 | 1059 | 2146 |
| Manson J.E. | MED | 9937 | 43 | 19.956 | 17 | 437 | 1769 |
| Nozik A.J. | CHEM | 2251 | 27 | 13.702 | 405 | 580 | 1488 |
| Kleinberg J. | CS | 2032 | 23 | 14.133 | 578 | 662 | 1282 |
| Halas N.J. | CHEM | 4928 | 35 | 17.596 | 64 | 299 | 2866 |
| Caruso F. | CHEM | 3024 | 22 | 14.786 | 171 | 780 | 2628 |
| Matsuzawa Y. | MED | 4584 | 31 | 13.709 | 153 | 352 | 2555 |
| Fuchs E. | MED | 2835 | 28 | 17.863 | 137 | 405 | 2477 |
| Mattick J.S. | BIO | 2933 | 29 | 14.975 | 241 | 418 | 1862 |
| Nolan S.P. | CHEM | 3679 | 26 | 16.584 | 187 | 246 | 3276 |
| Cohen P. | MED | 3173 | 24 | 14.811 | 284 | 491 | 1903 |
| Maldacena J. | PHYS | 1728 | 17 | 12.700 | 889 | 1096 | 1395 |
| Baylin S.B. | MED | 6578 | 37 | 13.268 | 60 | 479 | 2547 |
| Rizzolatti G. | BRAIN | 3671 | 31 | 15.504 | 30 | 1397 | 3037 |
| Carpenter S.R. | BIO | 3905 | 30 | 14.398 | 116 | 892 | 1422 |
| Reddy J.N. | ENG | 1759 | 19 | 15.250 | 501 | 797 | 1685 |
| Tamura K. | BIO | 9533 | 13 | 5.117 | 60 | 8744 | 9063 |
| Wilson P.W.F. | MED | 5760 | 32 | 13.960 | 54 | 860 | 2114 |
| Herrera F. | CS | 2989 | 31 | 17.060 | 53 | 914 | 2423 |
| Weissman I.L. | MED | 5485 | 34 | 16.754 | 112 | 169 | 2928 |
| Perou C.M. | MED | 7694 | 37 | 11.236 | 55 | 786 | 2151 |
| Lanier L.L. | INFDIS | 2919 | 25 | 13.997 | 376 | 517 | 1459 |
| Farrugia L.J. | CHEM | 2962 | 8 | 5.850 | 2746 | 2845 | 2870 |
| Kohn W. | PHYS | 3992 | 14 | 10.667 | 168 | 2161 | 3987 |
| Russell J.A. | SOC | 1511 | 17 | 13.583 | 769 | 1125 | 1396 |
| Mendel J.M. | CS | 1767 | 21 | 17.000 | 329 | 730 | 1630 |
| Peng F.Z. | CS | 2277 | 22 | 14.642 | 318 | 747 | 1719 |
| Schwartz J. | MED | 3712 | 22 | 15.702 | 229 | 543 | 1748 |
| Grant B.F. | HEALTH | 3897 | 28 | 13.651 | 67 | 1184 | 2347 |
| Kennicutt Jr. R.C. | PHYS | 2728 | 24 | 10.817 | 523 | 887 | 1265 |
| Tersoff J. | PHYS | 1708 | 20 | 13.986 | 619 | 984 | 1144 |
| List B. | CHEM | 1975 | 23 | 17.067 | 264 | 555 | 1937 |
| Nolen-Hoeksema S. | SOC | 1654 | 21 | 16.367 | 387 | 826 | 1377 |
| Maes M. | BRAIN | 2404 | 22 | 12.883 | 187 | 1398 | 2043 |
| Goodman R. | BRAIN | 1932 | 19 | 11.295 | 717 | 1087 | 1496 |
| Gallese V. | BRAIN | 2437 | 24 | 15.107 | 251 | 859 | 1318 |
| Sherr C.J. | MED | 1710 | 21 | 14.796 | 443 | 887 | 1374 |
| Wynn T.A. | INFDIS | 2193 | 21 | 12.920 | 495 | 734 | 1651 |
| Sirringhaus H. | PHYS | 2845 | 26 | 12.746 | 175 | 901 | 2037 |
| Rubinsztein D.C. | BRAIN | 4038 | 33 | 12.768 | 109 | 516 | 2496 |
| Nemeroff C.B. | BRAIN | 3336 | 28 | 15.646 | 144 | 462 | 2092 |
| He J.-H. | MATH | 1255 | 17 | 16.143 | 715 | 1026 | 1169 |
| Dolan R.J. | BRAIN | 5505 | 32 | 18.168 | 79 | 147 | 3921 |
| Bennett C.H. | PHYS | 2013 | 19 | 10.269 | 376 | 1919 | 1919 |
| Fischl B. | BRAIN | 4383 | 33 | 12.134 | 41 | 1459 | 2422 |
| Wolfe F. | MED | 3773 | 25 | 10.555 | 123 | 1454 | 2290 |
| Ross R. | MED | 1838 | 14 | 10.517 | 1128 | 1324 | 1668 |
| Ware Jr. J.E. | HEALTH | 4080 | 24 | 9.906 | 67 | 2381 | 3100 |
| Bazant Z.P. | ENG | 1804 | 17 | 13.833 | 348 | 1506 | 1694 |
| Campisi J. | MED | 2525 | 25 | 12.898 | 313 | 569 | 1938 |
| Zhao D. | CHEM | 6221 | 34 | 15.974 | 8 | 1245 | 4710 |
| Frechet J.M.J. | CHEM | 5356 | 31 | 18.000 | 81 | 152 | 3945 |
| Vogelstein B. | MED | 9556 | 48 | 18.838 | 2 | 930 | 4054 |
| Willner I. | CHEM | 4222 | 28 | 17.603 | 35 | 583 | 3951 |
| Drevets W.C. | BRAIN | 2491 | 25 | 15.921 | 191 | 714 | 1481 |
| Barone V. | CHEM | 4116 | 20 | 13.817 | 82 | 1065 | 3458 |
| Jack Jr. C.R. | BRAIN | 5577 | 32 | 11.956 | 40 | 1336 | 2253 |
| Edgar R.C. | BIO | 3134 | 10 | 5.291 | 2266 | 2512 | 2519 |
| Austin P.C. | MED | 2261 | 22 | 14.925 | 474 | 761 | 883 |
| Atwater H.A. | PHYS | 3659 | 29 | 15.139 | 56 | 774 | 2772 |
| Slatkin M. | BIO | 2168 | 20 | 13.549 | 591 | 809 | 1071 |
| Liz-Marzan L.M. | CHEM | 3478 | 27 | 16.554 | 151 | 316 | 2355 |
| Maeda H. | MED | 2451 | 19 | 11.066 | 349 | 1093 | 2295 |
| Israelachvili J.N. | CHEM | 2433 | 21 | 13.426 | 316 | 784 | 1766 |
| Zimmet P. | MED | 6532 | 35 | 10.412 | 57 | 648 | 3163 |
| Stern Y. | BRAIN | 3423 | 24 | 12.295 | 350 | 549 | 1520 |
| Heckman J.J. | SOC | 1598 | 22 | 15.533 | 294 | 1057 | 1287 |
| Berner R.A. | EARTH | 1364 | 20 | 15.983 | 530 | 830 | 1169 |
| Tokura Y. | PHYS | 4046 | 25 | 12.903 | 147 | 415 | 3491 |
| Li Y. | CHEM | 3724 | 27 | 11.999 | 247 | 439 | 2059 |
| Verkman A.S. | MED | 3270 | 23 | 15.319 | 199 | 362 | 2696 |
| Kaufman R.J. | MED | 3429 | 32 | 15.584 | 166 | 253 | 2091 |
| Haffner S.M. | MED | 3969 | 26 | 12.890 | 148 | 770 | 1687 |
| Folke C. | BIO | 3857 | 31 | 11.346 | 201 | 778 | 1166 |
| O'Neill L.A.J. | MED | 2336 | 25 | 14.612 | 210 | 656 | 1797 |
| Catterall W.A. | BRAIN | 2363 | 21 | 12.551 | 375 | 662 | 2049 |
| Pfurtscheller G. | CS | 2792 | 25 | 14.868 | 90 | 1081 | 2103 |
| Caspi A. | SOC | 4112 | 29 | 15.769 | 34 | 1308 | 2015 |
| Facchetti A. | CHEM | 3535 | 29 | 13.921 | 288 | 502 | 897 |
| Cohen J. | SOC | 1920 | 11 | 9.028 | 1618 | 1636 | 1842 |
| Balkwill F.R. | MED | 2332 | 22 | 11.752 | 327 | 848 | 1937 |
| Van Genuchten M.T. | ENG | 2032 | 15 | 11.600 | 867 | 963 | 1609 |
| Sabatini D.M. | MED | 5230 | 37 | 14.088 | 94 | 146 | 4039 |
| Brabec C.J. | CHEM | 3712 | 28 | 11.879 | 75 | 939 | 2986 |
| Blochl P.E. | CHEM | 2879 | 9 | 5.117 | 2418 | 2697 | 2806 |
| Clutton-Brock T.H. | BIO | 2082 | 20 | 13.910 | 336 | 935 | 1453 |
| Bork P. | BIO | 7977 | 38 | 14.923 | 49 | 266 | 2251 |
| Wu G. | MED | 2816 | 22 | 12.857 | 173 | 919 | 2198 |
| Blair S.N. | HEALTH | 5348 | 33 | 14.265 | 52 | 565 | 2181 |
| Beutler B. | MED | 2455 | 26 | 13.482 | 261 | 579 | 1585 |
| Rajkumar S.V. | MED | 4075 | 33 | 11.935 | 130 | 585 | 1619 |
| Mozaffarian D. | MED | 6031 | 28 | 12.335 | 60 | 1204 | 1562 |
| Acemoglu D. | SOC | 1541 | 18 | 13.750 | 321 | 1519 | 1525 |
| Dresselhaus M.S. | PHYS | 5247 | 32 | 13.911 | 22 | 1147 | 2894 |
| Wiens J.J. | BIO | 1531 | 21 | 15.837 | 285 | 936 | 1418 |
| Rao C.N.R. | CHEM | 4208 | 24 | 14.918 | 30 | 1171 | 3948 |
| Smyth G.K. | MED | 3101 | 20 | 9.089 | 618 | 833 | 1607 |
| Logothetis N.K. | BRAIN | 2317 | 23 | 13.942 | 235 | 749 | 1640 |
| Felsenstein J. | BIO | 1353 | 14 | 12.667 | 1075 | 1116 | 1339 |
| Damasio A.R. | BRAIN | 2426 | 27 | 14.391 | 207 | 461 | 1903 |
| Schluter D. | BIO | 1743 | 23 | 15.823 | 363 | 585 | 1238 |
| Fukuzumi S. | CHEM | 3331 | 20 | 13.954 | 130 | 848 | 2529 |
| Benowitz N.L. | HEALTH | 2289 | 20 | 13.494 | 300 | 878 | 1561 |
| Norskov J.K. | CHEM | 5160 | 37 | 16.928 | 16 | 546 | 3606 |
| Shulman G.I. | MED | 4807 | 37 | 14.661 | 113 | 154 | 2665 |
| Fidler I.J. | MED | 2010 | 20 | 13.366 | 439 | 652 | 1623 |
| Van Os J. | BRAIN | 4879 | 27 | 14.900 | 57 | 704 | 2161 |
| Hunter T. | MED | 2914 | 23 | 14.985 | 302 | 383 | 1557 |
| Van Der Aalst W.M.P. | CS | 2165 | 21 | 13.106 | 210 | 1097 | 1792 |
| Travis W.D. | MED | 3959 | 30 | 10.885 | 99 | 1131 | 1590 |
| Hench L.L. | HEALTH | 1909 | 20 | 13.979 | 348 | 789 | 1544 |
| Nielsen R. | BIO | 3222 | 32 | 13.828 | 139 | 548 | 1321 |
| Murphy C.J. | CHEM | 2924 | 30 | 15.175 | 68 | 667 | 2283 |
| Bland J.M. | MED | 3804 | 21 | 14.183 | 19 | 3066 | 3528 |
| Fonarow G.C. | MED | 4799 | 32 | 12.994 | 59 | 781 | 1768 |
| Heaney R.P. | MED | 2322 | 21 | 12.348 | 343 | 843 | 1457 |
| Rutgeerts P. | MED | 5815 | 38 | 12.555 | 28 | 682 | 2671 |
| Deyo R.A. | HEALTH | 3249 | 27 | 12.789 | 72 | 1219 | 1932 |
| Kalluri R. | MED | 2892 | 24 | 12.245 | 128 | 941 | 2218 |
| Tour J.M. | CHEM | 4162 | 31 | 15.883 | 104 | 179 | 3088 |
| Daniels S.R. | MED | 4850 | 32 | 13.815 | 134 | 447 | 1057 |
| Strogatz S.H. | MATH | 2913 | 18 | 11.053 | 413 | 553 | 2618 |
| Remuzzi G. | MED | 5472 | 31 | 13.813 | 62 | 426 | 2387 |
| Stanley H.E. | MATH | 4926 | 30 | 14.508 | 75 | 254 | 3472 |
| Serruys P.W. | MED | 9087 | 39 | 13.972 | 7 | 744 | 4378 |
| Quigley H.A. | MED | 2376 | 22 | 11.973 | 265 | 1041 | 1380 |
| Dang C.V. | MED | 2419 | 28 | 11.784 | 293 | 532 | 1488 |
| Deisseroth K. | BRAIN | 4433 | 39 | 13.299 | 97 | 227 | 2261 |
| Berridge M.J. | BRAIN | 1489 | 18 | 13.867 | 468 | 1091 | 1174 |
| Chisti Y. | BIO | 1778 | 17 | 10.950 | 791 | 865 | 1481 |
| Fehr E. | SOC | 2560 | 28 | 14.854 | 42 | 1408 | 2192 |
| Evans R.M. | MED | 4364 | 33 | 14.842 | 101 | 186 | 2868 |
| Peng X. | CHEM | 3192 | 30 | 15.596 | 58 | 504 | 2715 |
| Stice E. | SOC | 1838 | 22 | 14.350 | 168 | 1201 | 1501 |
| Bard A.J. | CHEM | 3513 | 23 | 17.000 | 93 | 351 | 3058 |
| Reich P.B. | BIO | 3854 | 27 | 14.833 | 56 | 951 | 1709 |
| Hewitt G.M. | BIO | 1483 | 17 | 11.642 | 859 | 864 | 1319 |
| Akaike H. | SOC | 2026 | 8 | 8.000 | 2018 | 2018 | 2024 |
| Ho Y.-S. | ENG | 2186 | 19 | 13.233 | 108 | 1853 | 2125 |
| Katsnelson M.I. | PHYS | 5312 | 28 | 12.851 | 141 | 553 | 1072 |
| Li H. | BIO | 4903 | 21 | 7.314 | 92 | 2729 | 2730 |
| Cohen S. | BRAIN | 2354 | 18 | 11.808 | 182 | 1642 | 1877 |
| Lloyd-Jones D.M. | MED | 5846 | 31 | 8.912 | 49 | 1604 | 1989 |
| Nakamura S. | PHYS | 2444 | 22 | 11.980 | 219 | 820 | 1889 |
| Hughes T.J.R. | ENG | 2377 | 23 | 14.801 | 118 | 918 | 1728 |
| Smith Jr. S.C. | MED | 9051 | 47 | 10.982 | 25 | 503 | 1991 |
| Pocock S.J. | MED | 4824 | 35 | 11.979 | 87 | 454 | 1695 |
| Clark A.G. | BIO | 4347 | 26 | 11.996 | 184 | 558 | 1300 |
| Schwartz S.H. | SOC | 1402 | 17 | 12.676 | 605 | 1021 | 1281 |
| Antzelevitch C. | MED | 2592 | 25 | 12.729 | 177 | 667 | 1734 |
| Grubbs R.H. | CHEM | 3462 | 25 | 15.369 | 146 | 234 | 2883 |
| Nicolaides K.H. | MED | 3876 | 22 | 12.924 | 156 | 380 | 3020 |
| Connolly S.J. | MED | 4733 | 35 | 9.222 | 27 | 1840 | 2660 |
| Kaelin Jr. W.G. | MED | 1959 | 22 | 13.986 | 326 | 549 | 1484 |
| Di Marzo V. | MED | 3181 | 22 | 13.935 | 159 | 579 | 1902 |
| Rader D.J. | MED | 4869 | 33 | 13.543 | 93 | 319 | 1816 |
| Cella D. | MED | 4168 | 27 | 12.618 | 77 | 862 | 1732 |
| Tononi G. | BRAIN | 2322 | 25 | 14.705 | 166 | 572 | 1639 |
| Insel T.R. | BRAIN | 1836 | 20 | 13.540 | 311 | 861 | 1388 |
| Scott J.F. | PHYS | 2548 | 18 | 12.810 | 351 | 603 | 1849 |
| Sawyers C.L. | MED | 3651 | 32 | 10.991 | 203 | 266 | 2277 |
| Anderson M.J. | BIO | 1783 | 16 | 9.463 | 738 | 1234 | 1563 |
| Mann J.J. | BRAIN | 3399 | 24 | 13.159 | 141 | 548 | 2014 |
| Balandin A.A. | PHYS | 2312 | 19 | 10.942 | 271 | 1084 | 1900 |
| Stampfer M.J. | MED | 9055 | 41 | 19.929 | 13 | 307 | 1715 |
| McGaugh J.L. | BRAIN | 1940 | 22 | 13.961 | 367 | 476 | 1474 |
| Kyle R.A. | MED | 4083 | 28 | 11.849 | 79 | 1077 | 1427 |
| Beven K. | EARTH | 1927 | 17 | 12.896 | 387 | 1028 | 1343 |
| Livermore D.M. | INFDIS | 2346 | 22 | 12.825 | 308 | 671 | 1272 |
| Wardle D.A. | BIO | 2673 | 23 | 13.403 | 122 | 821 | 1962 |
| Kandel E.R. | BRAIN | 2955 | 23 | 14.929 | 252 | 300 | 1741 |
| Talley N.J. | MED | 3937 | 26 | 12.993 | 91 | 697 | 1793 |
| Langlois D. | MED | 3598 | 19 | 12.699 | 141 | 1037 | 1696 |
| Voinnet O. | BIO | 2032 | 24 | 13.885 | 275 | 484 | 1511 |
| Katon W. | HEALTH | 3193 | 23 | 12.971 | 131 | 888 | 1501 |
| Pearson R.G. | CHEM | 1112 | 16 | 14.500 | 773 | 827 | 1106 |
| Bray G.A. | MED | 3175 | 23 | 10.740 | 316 | 748 | 1135 |
| Lefkowitz R.J. | MED | 3822 | 29 | 15.292 | 92 | 243 | 2707 |
| Sun Y. | CHEM | 2869 | 26 | 13.546 | 60 | 1422 | 1583 |
| Elliot A.J. | SOC | 1823 | 23 | 14.943 | 140 | 1012 | 1435 |
| Svergun D.I. | CHEM | 2412 | 20 | 11.403 | 324 | 713 | 1723 |
| Bhatt D.L. | MED | 4530 | 30 | 11.400 | 69 | 835 | 1696 |
| Levine R. | SOC | 1493 | 23 | 15.500 | 270 | 598 | 1324 |
| Baraniuk R.G. | CS | 2125 | 21 | 11.802 | 302 | 725 | 1691 |
| Trinchieri G. | INFDIS | 2203 | 24 | 12.994 | 387 | 520 | 1013 |
| Stephan D.W. | CHEM | 2105 | 24 | 14.979 | 209 | 384 | 1924 |
| Rice J.R. | ENG | 1418 | 21 | 14.667 | 312 | 727 | 1348 |
| Yusuf S. | MED | 2650 | 22 | 7.651 | 355 | 1297 | 1668 |
| Kantarjian H. | MED | 8541 | 39 | 13.887 | 4 | 1306 | 3206 |
| Elimelech M. | ENG | 4058 | 31 | 20.893 | 26 | 247 | 3401 |
| Feinberg A.P. | MED | 2301 | 24 | 12.426 | 202 | 640 | 1744 |
| Fama E.F. | SOC | 1672 | 19 | 12.333 | 185 | 1658 | 1658 |
| Thuiller W. | BIO | 2982 | 29 | 13.539 | 106 | 628 | 1436 |
| Elad M. | CS | 2699 | 24 | 13.700 | 141 | 736 | 1450 |
| Deb K. | CS | 2766 | 13 | 9.617 | 252 | 2130 | 2359 |
| Lips P. | MED | 2623 | 24 | 12.982 | 255 | 530 | 1256 |
| Chen C.-W. | ENG | 1666 | 21 | 17.360 | 279 | 598 | 976 |
| Shleifer A. | SOC | 3724 | 29 | 15.983 | 42 | 587 | 2073 |
| Oxman A.D. | HEALTH | 4241 | 32 | 10.785 | 212 | 461 | 936 |
| Henikoff S. | BIO | 3232 | 26 | 15.819 | 66 | 487 | 2324 |
| Schacter D.L. | BRAIN | 2704 | 21 | 14.393 | 114 | 692 | 2192 |
| Cannon C.P. | MED | 5498 | 35 | 11.760 | 51 | 777 | 1258 |
| Sporns O. | BRAIN | 2678 | 25 | 12.609 | 129 | 612 | 2116 |
| Legendre P. | BIO | 2430 | 23 | 13.517 | 167 | 706 | 1514 |
| Andreasen N.C. | BRAIN | 2117 | 21 | 12.544 | 253 | 842 | 1369 |
| Davis F.D. | SOC | 2574 | 11 | 6.821 | 1220 | 1263 | 2524 |
| Helbing D. | MATH | 1853 | 20 | 11.550 | 251 | 1122 | 1588 |
| MacKerell Jr. A.D. | CHEM | 3334 | 21 | 11.994 | 76 | 1274 | 2283 |
| Karplus M. | CHEM | 3860 | 22 | 14.713 | 87 | 353 | 3408 |
| Belytschko T. | ENG | 3440 | 25 | 17.128 | 16 | 1187 | 3209 |
| Olfati-Saber R. | CS | 1881 | 12 | 9.833 | 521 | 1854 | 1866 |
| Bullmore E.T. | BRAIN | 5331 | 37 | 16.245 | 8 | 771 | 3106 |
| Zahra S.A. | SOC | 1341 | 21 | 13.500 | 267 | 1067 | 1255 |
| Excoffier L. | BIO | 2854 | 22 | 12.718 | 45 | 1737 | 2541 |
| Manolio T.A. | MED | 3458 | 25 | 9.998 | 149 | 952 | 1540 |
| Lynch M. | BIO | 1668 | 19 | 13.273 | 294 | 962 | 1309 |
| Morris R.G.M. | BRAIN | 1703 | 18 | 12.368 | 412 | 775 | 1495 |
| Akagi H. | CS | 1805 | 19 | 13.583 | 273 | 713 | 1666 |
| Antman E.M. | MED | 6504 | 44 | 9.978 | 34 | 682 | 1585 |
| Gubler D.J. | INFDIS | 1737 | 20 | 14.526 | 426 | 560 | 1024 |
| Stadtman E.R. | BIO | 1894 | 20 | 14.247 | 273 | 573 | 1557 |
| Salzberg S.L. | BIO | 6517 | 35 | 11.722 | 59 | 211 | 3237 |
| Stamatakis A. | BIO | 2596 | 13 | 5.612 | 1121 | 1836 | 1962 |
| Giedd J.N. | BRAIN | 2980 | 28 | 11.771 | 138 | 675 | 1367 |
| Yablonovitch E. | PHYS | 1723 | 17 | 9.215 | 729 | 985 | 1450 |
| Cohen J.D. | BRAIN | 4813 | 34 | 15.326 | 22 | 379 | 3454 |
| Hyeon T. | CHEM | 3960 | 31 | 14.430 | 67 | 216 | 3335 |
| Chapin III F.S. | BIO | 4440 | 30 | 13.009 | 38 | 921 | 1703 |
| Coleman J.N. | CHEM | 3268 | 26 | 10.980 | 79 | 852 | 2432 |
| Clapham D.E. | MED | 1921 | 22 | 13.870 | 311 | 426 | 1522 |
| Mallat S. | CS | 1784 | 12 | 8.667 | 912 | 1538 | 1674 |
| Kerbel R.S. | MED | 2174 | 22 | 11.926 | 305 | 490 | 1747 |
| Camerer C.F. | SOC | 1982 | 23 | 12.904 | 264 | 678 | 1176 |
| Slovic P. | SOC | 1735 | 20 | 11.951 | 435 | 793 | 1092 |
| Barrett J.C. | MED | 6108 | 32 | 8.928 | 40 | 1319 | 1788 |
| Varki A. | INFDIS | 1787 | 18 | 13.700 | 428 | 532 | 1422 |
| Schapira A.H.V. | BRAIN | 2392 | 23 | 11.212 | 296 | 609 | 1358 |
| Pollak M. | MED | 2486 | 24 | 12.218 | 355 | 457 | 1053 |
| Kalivas P.W. | BRAIN | 2214 | 22 | 13.254 | 154 | 683 | 1842 |
| Faraone S.V. | BRAIN | 5612 | 29 | 13.606 | 23 | 828 | 2300 |
| Anderson K.C. | MED | 6531 | 33 | 13.316 | 58 | 159 | 3281 |
| Cooper C. | MED | 5819 | 33 | 13.779 | 26 | 599 | 1979 |
| Lindquist S. | BIO | 2974 | 27 | 15.918 | 107 | 241 | 2337 |
| Yates III J.R. | MED | 6044 | 31 | 14.401 | 45 | 309 | 2160 |
| Tschopp J. | MED | 5499 | 36 | 16.699 | 24 | 192 | 3618 |
| Delley B. | PHYS | 1546 | 14 | 9.016 | 1109 | 1152 | 1283 |
| Pearce J.A. | EARTH | 1546 | 19 | 11.250 | 351 | 1178 | 1267 |
| Anderson J.L. | MED | 5330 | 39 | 8.349 | 117 | 684 | 943 |
| Higgins E.T. | SOC | 1352 | 20 | 13.976 | 415 | 634 | 1157 |
| Janiak C. | CHEM | 1745 | 18 | 11.122 | 483 | 684 | 1593 |
| Blagosklonny M.V. | MED | 1698 | 19 | 12.771 | 492 | 652 | 1049 |
| Mann S. | CHEM | 2327 | 22 | 12.957 | 227 | 402 | 2019 |
| Mulvaney P. | CHEM | 2658 | 25 | 13.566 | 224 | 289 | 1687 |
| Fava M. | BRAIN | 3293 | 25 | 11.067 | 141 | 612 | 1741 |
| Ron D. | MED | 3963 | 32 | 13.875 | 37 | 429 | 2792 |
| Astruc D. | CHEM | 2261 | 16 | 12.017 | 279 | 718 | 2180 |
| Bonventre J.V. | MED | 3268 | 28 | 12.912 | 133 | 381 | 1582 |
| Stone G.W. | MED | 5465 | 34 | 10.933 | 10 | 1586 | 3232 |
| Taylor S.E. | SOC | 1664 | 20 | 14.694 | 179 | 933 | 1314 |
| Emanuel K.A. | EARTH | 1300 | 18 | 14.767 | 514 | 636 | 983 |
| Livak K.J. | BIO | 7462 | 11 | 5.017 | 70 | 5680 | 6574 |
| Barrett-Connor E. | MED | 4771 | 24 | 12.945 | 103 | 528 | 1395 |
| Kishimoto T. | MED | 3303 | 27 | 12.993 | 174 | 255 | 1847 |
| Hall A. | MED | 2254 | 22 | 13.660 | 265 | 327 | 1813 |
| Lipton S.A. | BRAIN | 2831 | 25 | 12.543 | 180 | 394 | 1710 |
| Moss A.J. | MED | 3664 | 27 | 10.141 | 85 | 1103 | 1487 |
| Antonietti M. | CHEM | 5656 | 36 | 20.336 | 8 | 363 | 2973 |
| Kimura M. | BIO | 1542 | 10 | 9.000 | 1321 | 1469 | 1541 |
| Jorm A.F. | HEALTH | 2428 | 18 | 13.207 | 242 | 828 | 1191 |
| Simon R. | MED | 2204 | 23 | 12.607 | 252 | 562 | 1236 |
| Korner C. | BIO | 1672 | 18 | 13.829 | 359 | 618 | 1341 |
| Nicholson J.K. | CHEM | 4238 | 29 | 13.982 | 21 | 877 | 2606 |
| Webster R.G. | INFDIS | 4384 | 26 | 12.950 | 59 | 519 | 2284 |
| Roger V.L. | MED | 5625 | 25 | 8.741 | 28 | 2148 | 2687 |
| Gale P.A. | CHEM | 1626 | 20 | 13.744 | 328 | 591 | 1278 |
| Bers D.M. | MED | 2104 | 19 | 12.891 | 353 | 489 | 1471 |
| Shine R. | BIO | 2372 | 16 | 11.983 | 312 | 614 | 2046 |
| Cuervo A.M. | MED | 3025 | 28 | 14.010 | 129 | 407 | 1253 |
| Dickson D.W. | BRAIN | 6419 | 33 | 11.916 | 86 | 351 | 1135 |
| Khosla S. | MED | 2582 | 25 | 12.884 | 129 | 632 | 1461 |
| Broderick J.P. | BRAIN | 2961 | 25 | 10.716 | 187 | 702 | 1233 |
| Pardridge W.M. | CHEM | 1457 | 16 | 13.138 | 517 | 658 | 1372 |
| Baltimore D. | INFDIS | 3754 | 31 | 15.537 | 66 | 153 | 3365 |
| Guengerich F.P. | MED | 2306 | 16 | 10.945 | 442 | 679 | 1595 |
| Kumar S. | BIO | 9757 | 17 | 8.986 | 23 | 913 | 9486 |
| Geschwind D.H. | BRAIN | 4453 | 30 | 12.883 | 116 | 317 | 1261 |
| Druker B.J. | MED | 3343 | 29 | 10.251 | 116 | 719 | 1391 |
| Moncada S. | MED | 2933 | 24 | 14.173 | 47 | 838 | 2302 |
| Lande R. | BIO | 1171 | 17 | 15.167 | 515 | 687 | 946 |
| Easton D.F. | MED | 5829 | 34 | 10.964 | 79 | 363 | 1443 |
| Stratton M.R. | MED | 6117 | 41 | 7.974 | 94 | 337 | 1818 |
| Klimov V.I. | CHEM | 2101 | 24 | 12.938 | 148 | 579 | 1726 |
| Coleman R.E. | MED | 2167 | 23 | 10.942 | 271 | 725 | 1175 |
| Thun M.J. | MED | 7794 | 39 | 13.772 | 6 | 451 | 4789 |
| Loewenstein G. | SOC | 2082 | 23 | 15.310 | 173 | 549 | 1087 |
| Breslau N. | HEALTH | 1794 | 22 | 12.699 | 145 | 1086 | 1343 |
| Friederici A.D. | BRAIN | 2018 | 19 | 13.529 | 239 | 587 | 1541 |
| Moller A.P. | BIO | 2108 | 16 | 10.792 | 364 | 862 | 1635 |
| Chertow G.M. | MED | 3881 | 28 | 11.559 | 59 | 758 | 1772 |
| Peto R. | MED | 5222 | 37 | 12.100 | 35 | 379 | 2247 |
| Karsenty G. | MED | 3043 | 28 | 13.204 | 110 | 294 | 2171 |
| Paterson D.L. | INFDIS | 3030 | 24 | 12.841 | 116 | 597 | 1477 |
| Ronquist F. | BIO | 4041 | 14 | 7.417 | 107 | 2370 | 3738 |
| Aurbach D. | ENG | 2571 | 24 | 10.870 | 94 | 924 | 2103 |
| Appel L.J. | HEALTH | 3969 | 31 | 9.944 | 105 | 663 | 1230 |
| Lok A.S.F. | MED | 2845 | 25 | 11.050 | 68 | 1059 | 2039 |
| Simberloff D. | BIO | 1737 | 19 | 13.920 | 344 | 604 | 1043 |
| Pumera M. | CHEM | 1511 | 16 | 13.600 | 432 | 649 | 1332 |
| Royston P. | MED | 1760 | 22 | 13.515 | 260 | 636 | 1030 |
| Paus T. | BRAIN | 2021 | 22 | 12.449 | 286 | 619 | 1033 |
| Holsboer F. | BRAIN | 3879 | 25 | 12.878 | 186 | 302 | 1242 |
| Sibai B.M. | MED | 2418 | 18 | 12.258 | 255 | 735 | 1303 |
| Meltzer H.Y. | BRAIN | 2659 | 21 | 12.955 | 173 | 614 | 1331 |
| Hortobagyi G.N. | MED | 5543 | 31 | 12.905 | 90 | 216 | 1633 |
| Nel A.E. | MED | 3102 | 25 | 9.710 | 69 | 1032 | 2527 |
| Wise R.A. | BRAIN | 1238 | 19 | 13.410 | 447 | 666 | 1045 |
| Schubert U.S. | CHEM | 4128 | 25 | 15.392 | 56 | 217 | 3597 |
| Singer W. | BRAIN | 2315 | 23 | 13.855 | 211 | 313 | 1655 |
| McKay G. | ENG | 2817 | 22 | 15.833 | 88 | 327 | 2560 |
| Van Essen D.C. | BRAIN | 2337 | 23 | 12.332 | 194 | 573 | 1290 |
| Liu Y.-J. | INFDIS | 3023 | 29 | 12.749 | 152 | 254 | 1655 |
| Carrera E. | ENG | 1245 | 18 | 13.352 | 401 | 837 | 1022 |
| Jellinger K. | BRAIN | 1692 | 18 | 12.801 | 473 | 693 | 850 |
| Poldrack R.A. | BRAIN | 1995 | 23 | 13.896 | 194 | 465 | 1346 |
| Van De Walle C.G. | PHYS | 1843 | 19 | 12.798 | 248 | 609 | 1585 |
| Welton T. | CHEM | 1754 | 16 | 8.563 | 784 | 785 | 1496 |
| McCrae R.R. | SOC | 1842 | 23 | 13.737 | 92 | 942 | 1584 |
| Knothe G. | ENG | 914 | 18 | 14.500 | 515 | 794 | 874 |
| Tabas I. | MED | 2232 | 22 | 12.250 | 217 | 516 | 1445 |
| Ricklefs R.E. | BIO | 1621 | 18 | 12.391 | 393 | 692 | 1155 |
| Go A.S. | MED | 7228 | 34 | 9.053 | 12 | 1694 | 2300 |
| Schatz G.C. | CHEM | 5030 | 31 | 16.554 | 62 | 134 | 2111 |
| Zeuzem S. | MED | 5763 | 31 | 11.263 | 36 | 595 | 1916 |
| Von Heijne G. | BIO | 3381 | 22 | 13.736 | 229 | 273 | 1270 |
| Aebersold R. | BIO | 6180 | 38 | 16.398 | 7 | 357 | 3707 |
| Dixon R.A. | BIO | 2789 | 23 | 13.075 | 101 | 578 | 1718 |
| Draine B.T. | PHYS | 1713 | 22 | 12.777 | 223 | 749 | 1060 |
| Green M.F. | BRAIN | 2128 | 22 | 10.817 | 207 | 826 | 1278 |
| Davies M.J. | MED | 1826 | 19 | 14.314 | 249 | 512 | 1315 |
| Zheng Y.-F. | EARTH | 1887 | 23 | 13.936 | 171 | 703 | 970 |
| Hernquist L. | PHYS | 2961 | 24 | 15.948 | 145 | 201 | 1713 |
| Moskovits M. | CHEM | 1697 | 18 | 10.977 | 460 | 601 | 1367 |
| Rice-Evans C. | MED | 2908 | 22 | 10.945 | 66 | 956 | 2581 |
| Marik P.E. | MED | 1505 | 17 | 12.562 | 233 | 1110 | 1334 |
| Camm A.J. | MED | 6179 | 36 | 9.960 | 16 | 1064 | 2065 |
| Haykin S. | CS | 1567 | 12 | 8.700 | 952 | 1131 | 1484 |
| Nusse R. | MED | 2534 | 24 | 13.827 | 146 | 280 | 2005 |
| Corrigan P.W. | HEALTH | 1548 | 20 | 12.594 | 196 | 983 | 1227 |
| Snyder S.H. | BRAIN | 3756 | 28 | 15.510 | 67 | 165 | 2823 |
| Rex D.K. | MED | 2276 | 23 | 10.763 | 128 | 933 | 1539 |
| Jin R. | CHEM | 2186 | 23 | 12.458 | 135 | 556 | 1759 |
| Ransohoff R.M. | BRAIN | 2641 | 23 | 13.534 | 124 | 488 | 1477 |
| Charney D.S. | BRAIN | 4340 | 27 | 14.213 | 89 | 197 | 2023 |
| Armand M. | ENG | 3209 | 21 | 10.026 | 73 | 1142 | 2328 |
| Califf R.M. | MED | 5971 | 33 | 11.211 | 46 | 317 | 2158 |
| Costerton J.W. | INFDIS | 2954 | 19 | 11.766 | 89 | 949 | 2035 |
| Surh Y.-J. | MED | 1792 | 18 | 13.086 | 261 | 573 | 1506 |
| Cook N.R. | MED | 2796 | 26 | 11.875 | 182 | 525 | 907 |
| Nie S. | CHEM | 3846 | 30 | 14.406 | 14 | 688 | 3258 |
| Bakker A.B. | SOC | 2485 | 23 | 14.860 | 61 | 926 | 1316 |
| Emery P. | MED | 6033 | 36 | 11.968 | 28 | 460 | 1689 |
| Blaser M.J. | MED | 2815 | 23 | 12.689 | 147 | 410 | 1588 |
| Kanwisher N. | BRAIN | 1957 | 23 | 14.933 | 97 | 526 | 1782 |
| Pyykko P. | CHEM | 1173 | 16 | 12.180 | 539 | 874 | 1033 |
| Brown J.H. | BIO | 2491 | 24 | 12.924 | 110 | 661 | 1286 |
| Arnsten A.F.T. | BRAIN | 1271 | 20 | 13.924 | 303 | 616 | 1105 |
| Tucker C.J. | EARTH | 2127 | 21 | 10.908 | 293 | 694 | 1036 |
| Baldocchi D.D. | BIO | 2510 | 25 | 11.220 | 221 | 596 | 884 |
| Oberdorster G. | MED | 1971 | 20 | 9.917 | 206 | 1148 | 1358 |
| Wong T.Y. | MED | 5112 | 27 | 12.888 | 19 | 825 | 2451 |
| Mayberg H.S. | BRAIN | 2198 | 29 | 10.574 | 178 | 573 | 1099 |
| Zlokovic B.V. | BRAIN | 1759 | 20 | 11.681 | 337 | 471 | 1465 |
| Shane S. | SOC | 1218 | 17 | 13.533 | 343 | 855 | 1079 |
| Bush A.I. | BRAIN | 2666 | 27 | 12.654 | 147 | 311 | 1523 |
| Barro R.J. | SOC | 966 | 17 | 13.500 | 462 | 871 | 962 |
| Hyman B.T. | BRAIN | 5221 | 32 | 13.357 | 44 | 289 | 1830 |
| Fahrig L. | BIO | 1411 | 18 | 11.810 | 457 | 652 | 1131 |
| Rush A.J. | BRAIN | 3911 | 28 | 10.364 | 53 | 867 | 1655 |
| Barkley R.A. | BRAIN | 1309 | 17 | 11.963 | 408 | 921 | 1051 |
| Hameed B.H. | ENG | 1907 | 21 | 16.000 | 87 | 594 | 1765 |
| Lipton R.B. | BRAIN | 3770 | 25 | 14.872 | 30 | 651 | 2098 |
| Tinetti M.E. | BRAIN | 1670 | 20 | 11.801 | 175 | 985 | 1389 |
| Barsalou L.W. | BRAIN | 1092 | 16 | 12.676 | 652 | 719 | 955 |
| Despres J.-P. | MED | 2986 | 22 | 10.942 | 144 | 693 | 1375 |
| Zhang Y. | BIO | 1513 | 18 | 12.108 | 346 | 622 | 1384 |
| Fauci A.S. | INFDIS | 3342 | 24 | 12.923 | 89 | 323 | 2417 |
| Stoddart J.F. | CHEM | 4307 | 31 | 15.539 | 86 | 100 | 2286 |
| Sullivan P.F. | BRAIN | 2894 | 24 | 11.889 | 154 | 629 | 962 |
| Goldhaber S.Z. | MED | 2800 | 25 | 11.406 | 121 | 544 | 1503 |
| Lewis N.S. | CHEM | 2420 | 19 | 10.872 | 215 | 580 | 1779 |
| Dickinson E. | CHEM | 1109 | 15 | 14.700 | 505 | 700 | 954 |
| Steriade M. | BRAIN | 1327 | 19 | 12.900 | 252 | 797 | 1248 |
| Gaston K.J. | BIO | 2715 | 22 | 11.793 | 117 | 552 | 1884 |
| Craig A.D. | BRAIN | 1083 | 13 | 11.286 | 916 | 950 | 1006 |
| Pollard T.D. | INFDIS | 1787 | 19 | 13.105 | 188 | 632 | 1545 |
| Tuomilehto J. | MED | 7457 | 39 | 11.670 | 13 | 583 | 1893 |
| Ramsey C.B. | EARTH | 2428 | 17 | 8.903 | 618 | 753 | 907 |
| Schwartz P.J. | MED | 3035 | 25 | 11.756 | 97 | 673 | 1264 |
| Mayer R.E. | SOC | 1255 | 19 | 13.600 | 273 | 734 | 1124 |
| Shahidi F. | CHEM | 2253 | 20 | 15.110 | 71 | 652 | 2048 |
| Fauser B.C.J.M. | MED | 2285 | 23 | 10.778 | 200 | 592 | 1312 |
| Porter M.E. | SOC | 1109 | 16 | 12.167 | 505 | 894 | 1033 |
| May R.M. | BIO | 1716 | 21 | 14.904 | 217 | 400 | 1252 |
| O'Doherty J.P. | BRAIN | 2205 | 27 | 13.041 | 118 | 555 | 1094 |
| Krum H. | MED | 3332 | 26 | 9.774 | 164 | 605 | 1069 |
| Ridley A.J. | MED | 1824 | 17 | 11.902 | 256 | 712 | 1535 |
| Hutmacher D.W. | HEALTH | 1956 | 19 | 11.486 | 313 | 571 | 1251 |
| Hutchings G.J. | CHEM | 2580 | 22 | 11.553 | 167 | 350 | 2214 |
| Fenech M. | HEALTH | 1476 | 18 | 10.799 | 402 | 761 | 1222 |
| Bradley M.M. | BRAIN | 1774 | 22 | 11.080 | 300 | 704 | 915 |
| Blair R.J.R. | BRAIN | 1232 | 20 | 14.983 | 311 | 521 | 960 |
| McGorry P.D. | BRAIN | 2955 | 25 | 11.866 | 113 | 433 | 1646 |
| Levin S.A. | BIO | 1898 | 20 | 12.764 | 292 | 417 | 1286 |
| Thompson S.G. | MED | 5418 | 27 | 12.953 | 38 | 381 | 2189 |
| Prior R.L. | CHEM | 2678 | 25 | 13.817 | 37 | 721 | 2315 |
| Inouye S.K. | BRAIN | 1861 | 22 | 11.826 | 149 | 768 | 1400 |
| Kennedy J. | CS | 3950 | 10 | 6.833 | 143 | 2844 | 3863 |
| Molnar P. | EARTH | 1779 | 21 | 13.506 | 135 | 751 | 1300 |
| Mannucci P.M. | MED | 3009 | 19 | 11.270 | 168 | 521 | 1781 |
| Shevach E.M. | INFDIS | 1788 | 22 | 12.519 | 310 | 381 | 1166 |
| Davis R.J. | MED | 2906 | 23 | 13.822 | 204 | 219 | 1404 |
| Zanchetti A. | MED | 3559 | 23 | 8.480 | 192 | 363 | 2549 |
| Reetz M.T. | CHEM | 1528 | 16 | 11.700 | 244 | 1064 | 1433 |
| Ceriello A. | MED | 1990 | 20 | 11.288 | 263 | 766 | 969 |
| Rogers J.A. | CHEM | 4412 | 31 | 12.902 | 21 | 424 | 3209 |
| Smith D.R. | PHYS | 4586 | 29 | 12.813 | 9 | 1149 | 3037 |
| Burton D.R. | INFDIS | 3842 | 32 | 11.296 | 51 | 525 | 1533 |
| Baker D. | BIO | 3925 | 31 | 14.972 | 57 | 153 | 2442 |
| Devarajan P. | MED | 2669 | 28 | 12.208 | 210 | 264 | 1079 |
| Marks T.J. | CHEM | 5188 | 32 | 16.889 | 29 | 102 | 3869 |
| Walsh C.T. | BIO | 2644 | 18 | 12.381 | 187 | 415 | 1945 |
| Gonzalez F.J. | MED | 4465 | 25 | 13.983 | 98 | 207 | 1693 |
| Loscher W. | BRAIN | 1725 | 19 | 12.975 | 187 | 698 | 1355 |
| Suslick K.S. | CHEM | 1819 | 21 | 13.485 | 138 | 535 | 1702 |
| Anderson P.W. | PHYS | 1158 | 16 | 12.417 | 538 | 712 | 1013 |
| Deeks S.G. | INFDIS | 2894 | 27 | 10.954 | 148 | 478 | 1098 |
| Corbetta M. | BRAIN | 2933 | 27 | 10.681 | 41 | 1075 | 1950 |
| Sandelowski M. | HEALTH | 1049 | 14 | 11.533 | 720 | 919 | 997 |
| Tomasello M. | BRAIN | 2456 | 21 | 14.857 | 58 | 540 | 2397 |
| Kollef M.H. | MED | 2127 | 21 | 11.384 | 151 | 593 | 1809 |
| Hawker C.J. | CHEM | 3654 | 26 | 13.604 | 34 | 579 | 2077 |
| Wyman C.E. | BIO | 2117 | 23 | 14.404 | 143 | 332 | 1540 |
| Albert R. | BIO | 3649 | 17 | 9.552 | 53 | 1488 | 3060 |
| Pollard J.W. | MED | 2404 | 23 | 10.616 | 280 | 295 | 1642 |
| De Lange T. | MED | 1659 | 22 | 13.504 | 259 | 287 | 1559 |
| Brenner H. | MED | 3807 | 23 | 11.990 | 44 | 813 | 1926 |
| Petri M. | MED | 3212 | 25 | 10.445 | 147 | 542 | 1136 |
| Wilczek F. | PHYS | 1408 | 20 | 14.167 | 290 | 369 | 1340 |
| Altieri D.C. | MED | 1611 | 19 | 12.982 | 338 | 381 | 1352 |
| Evans A.C. | BRAIN | 5646 | 39 | 17.262 | 34 | 70 | 2533 |
| Blackburn E.H. | MED | 1697 | 21 | 12.074 | 318 | 431 | 1171 |
| Pascual-Leone A. | BRAIN | 4572 | 31 | 15.465 | 10 | 575 | 2753 |
| Cuijpers P. | HEALTH | 2359 | 22 | 12.904 | 82 | 867 | 1322 |
| Hutchinson J.W. | ENG | 2452 | 21 | 14.850 | 112 | 338 | 1843 |
| Scrosati B. | ENG | 3285 | 22 | 12.702 | 103 | 395 | 1751 |
| Holt R.D. | BIO | 2108 | 21 | 13.759 | 178 | 469 | 1129 |
| Hanauer S.B. | MED | 3028 | 29 | 10.498 | 108 | 551 | 1135 |
| Berwick D.M. | HEALTH | 1199 | 18 | 12.636 | 375 | 747 | 949 |
| Springer T.A. | INFDIS | 2264 | 20 | 13.300 | 215 | 272 | 1821 |
| Price C.J. | BRAIN | 1932 | 21 | 12.332 | 205 | 518 | 1261 |
| Wazwaz A.-M. | MATH | 1014 | 12 | 11.500 | 904 | 963 | 1008 |
| Issa J.-P.J. | MED | 2600 | 25 | 11.903 | 144 | 342 | 1579 |
| Bredas J.L. | CHEM | 4026 | 26 | 13.983 | 28 | 556 | 2126 |
| Cummings S.R. | MED | 4758 | 30 | 12.673 | 12 | 833 | 2658 |
| Bax A. | BRAIN | 2818 | 22 | 14.796 | 56 | 397 | 2560 |
| Bader R.F.W. | CHEM | 1123 | 16 | 12.117 | 455 | 816 | 1069 |
| Haldane F.D.M. | PHYS | 901 | 15 | 13.750 | 620 | 727 | 879 |
| Goldberg A.L. | BIO | 2915 | 25 | 13.600 | 127 | 199 | 1973 |
| Wenzel S.E. | MED | 2339 | 24 | 9.934 | 220 | 548 | 1177 |
| Glover G.H. | BRAIN | 3176 | 29 | 14.246 | 91 | 350 | 921 |
| Pedrycz W. | CS | 1713 | 17 | 12.810 | 267 | 610 | 1267 |
| Goadsby P.J. | BRAIN | 2295 | 21 | 11.609 | 141 | 522 | 1805 |
| Cullen B.R. | INFDIS | 1699 | 23 | 14.069 | 228 | 239 | 1608 |
| Lassmann H. | BRAIN | 2894 | 25 | 11.529 | 147 | 351 | 1434 |
| Bellomo R. | MED | 4608 | 28 | 11.842 | 22 | 850 | 1951 |
| Kahn C.R. | MED | 4455 | 31 | 14.956 | 60 | 105 | 2632 |
| Diez Roux A.V. | HEALTH | 2265 | 20 | 12.846 | 277 | 541 | 716 |
| Portner H.O. | BIO | 1544 | 17 | 12.868 | 281 | 658 | 1184 |
| Nocera D.G. | CHEM | 2861 | 25 | 13.679 | 139 | 152 | 2290 |
| Simopoulos A.P. | HEALTH | 919 | 13 | 12.333 | 822 | 892 | 894 |
| Reik W. | MED | 2615 | 27 | 10.415 | 127 | 444 | 1554 |
| Javitt D.C. | BRAIN | 2010 | 21 | 12.865 | 187 | 491 | 1202 |
| Dorenbos P. | PHYS | 1270 | 15 | 14.067 | 526 | 617 | 803 |
| Hawking S.W. | PHYS | 1079 | 16 | 12.833 | 488 | 665 | 1034 |
| Strachan D.P. | MED | 4636 | 32 | 9.041 | 228 | 342 | 603 |
| Bejan A. | ENG | 1409 | 14 | 12.000 | 395 | 777 | 1340 |
| Schulz K.F. | MED | 4213 | 31 | 12.485 | 17 | 1102 | 1424 |
| Cloninger C.R. | BRAIN | 1971 | 20 | 10.876 | 278 | 693 | 944 |
| Zhang S. | CHEM | 1691 | 21 | 12.737 | 244 | 450 | 1176 |
| Prentice R.L. | MED | 2651 | 25 | 9.793 | 197 | 581 | 981 |
| Bridgwater A.V. | ENG | 1342 | 18 | 11.253 | 437 | 647 | 1039 |
| Ebbesen T.W. | CS | 3545 | 24 | 11.851 | 31 | 776 | 2589 |
| Glaeser E.L. | SOC | 1339 | 21 | 15.367 | 95 | 941 | 1144 |
| Zurek W.H. | PHYS | 1253 | 15 | 12.708 | 502 | 553 | 1199 |
| Kollman P.A. | CHEM | 3610 | 23 | 11.132 | 83 | 349 | 2619 |
| Manthiram A. | ENG | 2311 | 24 | 16.542 | 88 | 212 | 2062 |
| Lord C. | SOC | 3390 | 26 | 11.714 | 32 | 1028 | 1668 |
| Ohno H. | PHYS | 2392 | 22 | 9.076 | 275 | 491 | 1411 |
| Callaway R.M. | BIO | 1975 | 23 | 11.976 | 123 | 613 | 1429 |
| Buchwald S.L. | CHEM | 4509 | 30 | 21.133 | 13 | 121 | 4423 |
| Costanza R. | SOC | 2234 | 20 | 10.589 | 139 | 1014 | 1228 |
| Pommier Y. | MED | 2579 | 18 | 11.389 | 197 | 486 | 1631 |
| Kahn S.E. | MED | 2506 | 23 | 9.879 | 151 | 714 | 1266 |
| Lupski J.R. | MED | 3120 | 26 | 11.968 | 113 | 274 | 1742 |
| Young R.A. | MED | 3935 | 34 | 10.859 | 104 | 121 | 2497 |
| Malenka R.C. | BRAIN | 3008 | 25 | 15.969 | 38 | 386 | 2051 |
| Murphy M.P. | MED | 2028 | 20 | 10.717 | 335 | 497 | 1041 |
| Van Cutsem E. | MED | 5766 | 35 | 9.937 | 9 | 1374 | 2041 |
| Adger W.N. | SOC | 1293 | 18 | 10.875 | 378 | 818 | 1023 |
| Braak H. | BRAIN | 3013 | 27 | 13.529 | 7 | 2060 | 2564 |
| Bunzli J.-C.G. | CHEM | 1850 | 16 | 10.893 | 283 | 727 | 1419 |
| Dimmeler S. | MED | 4169 | 32 | 13.356 | 15 | 489 | 2774 |
| Birbaumer N. | BRAIN | 3526 | 27 | 12.895 | 67 | 362 | 1498 |
| White H.D. | MED | 4529 | 32 | 9.972 | 71 | 353 | 1503 |
| Lakowicz J.R. | CHEM | 1928 | 17 | 11.759 | 214 | 653 | 1471 |
| Laird P.W. | MED | 3826 | 25 | 9.853 | 220 | 309 | 1068 |
| Kim V.N. | BIO | 2148 | 21 | 9.277 | 200 | 558 | 1911 |
| Elledge S.J. | MED | 4449 | 34 | 15.352 | 41 | 95 | 2937 |
| Grace A.A. | BRAIN | 1774 | 21 | 14.351 | 148 | 382 | 1518 |
| Giannelis E.P. | CHEM | 1888 | 23 | 13.853 | 203 | 236 | 1499 |
| Henrissat B. | BIO | 4144 | 32 | 10.226 | 99 | 329 | 1127 |
| Lakatta E.G. | MED | 3796 | 26 | 11.639 | 110 | 344 | 1187 |
| Russell T.P. | CHEM | 3893 | 27 | 14.822 | 63 | 157 | 2297 |
| Reid I.R. | MED | 2212 | 21 | 10.319 | 170 | 619 | 1490 |
| Flier J.S. | MED | 2940 | 28 | 13.985 | 101 | 159 | 1934 |
| Sontag E.D. | CS | 1361 | 19 | 12.583 | 359 | 520 | 957 |
| Lukin M.D. | PHYS | 3241 | 29 | 13.146 | 61 | 249 | 2041 |
| Seligman M.E.P. | SOC | 1557 | 18 | 13.374 | 144 | 724 | 1498 |
| DeLong E.F. | BIO | 1744 | 22 | 12.205 | 198 | 416 | 1387 |
| Running S.W. | EARTH | 3019 | 26 | 13.690 | 86 | 262 | 1634 |
| Zhang L. | CHEM | 2920 | 22 | 12.691 | 52 | 718 | 1773 |
| Holtzman D.M. | BRAIN | 4235 | 29 | 13.862 | 42 | 262 | 1892 |
| Chen G. | MATH | 4763 | 31 | 18.883 | 6 | 389 | 3018 |
| Blennow K. | BRAIN | 4181 | 26 | 11.086 | 51 | 590 | 1531 |
| Fulda S. | MED | 1697 | 18 | 12.331 | 198 | 761 | 1129 |
| Zettl A. | PHYS | 3168 | 27 | 11.917 | 159 | 172 | 1616 |
| Hausdorff J.M. | BRAIN | 2197 | 22 | 12.938 | 99 | 616 | 1299 |
| Naatanen R. | BRAIN | 1954 | 18 | 11.951 | 131 | 761 | 1647 |
| Hunter C.A. | CHEM | 2333 | 20 | 10.663 | 179 | 661 | 1225 |
| Saper C.B. | BRAIN | 2398 | 25 | 12.871 | 90 | 480 | 1300 |
| MacDiarmid A.G. | CHEM | 1858 | 22 | 12.345 | 211 | 405 | 1179 |
| Lang F. | MED | 3639 | 25 | 12.490 | 40 | 441 | 2396 |
| Kanatzidis M.G. | CHEM | 3493 | 23 | 11.861 | 105 | 201 | 2760 |
| Dekker C. | PHYS | 2361 | 25 | 12.995 | 157 | 175 | 1995 |
| Ternes T.A. | ENG | 1895 | 21 | 11.172 | 194 | 560 | 1283 |
| Valiev R.Z. | ENG | 2296 | 20 | 10.788 | 90 | 1015 | 1585 |
| Liaw Y.-F. | MED | 2098 | 20 | 10.726 | 140 | 815 | 1375 |
| Zhang Z. | CS | 1224 | 12 | 9.250 | 909 | 972 | 1128 |
| Diener H.-C. | BRAIN | 5262 | 28 | 9.891 | 47 | 654 | 1312 |
| Yu J. | CHEM | 4865 | 36 | 21.522 | 0 | 1858 | 2323 |
| Kenyon C. | MED | 1435 | 19 | 12.519 | 279 | 424 | 1393 |
| Giorgi F. | EARTH | 1827 | 21 | 12.680 | 137 | 671 | 1142 |
| Delmas P.D. | MED | 3754 | 26 | 11.955 | 77 | 328 | 1594 |
| Wang J. | CS | 3505 | 26 | 10.984 | 43 | 718 | 1746 |
| Coey J.M.D. | PHYS | 1606 | 17 | 11.383 | 207 | 809 | 1429 |
| Rosendaal F.R. | MED | 3424 | 24 | 12.782 | 97 | 316 | 1410 |
| Rousset F. | BIO | 1467 | 13 | 8.452 | 816 | 904 | 1200 |
| Glass C.K. | MED | 4140 | 34 | 14.104 | 14 | 506 | 1937 |
| Feringa B.L. | CHEM | 3071 | 21 | 14.824 | 84 | 236 | 2219 |
| Collins J.J. | MED | 3589 | 31 | 15.277 | 19 | 347 | 2378 |
| Gordon J.I. | MED | 7160 | 40 | 14.632 | 21 | 50 | 5040 |
| Allen F.H. | CHEM | 1571 | 12 | 6.510 | 887 | 1382 | 1464 |
| Roux B. | CHEM | 3113 | 20 | 13.783 | 137 | 272 | 1493 |
| Hanski I. | BIO | 1406 | 17 | 11.039 | 312 | 742 | 1205 |
| Hollis B.W. | MED | 2587 | 22 | 12.535 | 194 | 467 | 734 |
| Owen A.M. | BRAIN | 2166 | 23 | 11.508 | 98 | 639 | 1475 |
| Bouchard C. | MED | 3732 | 24 | 10.995 | 83 | 393 | 1749 |
| Suresh S. | ENG | 1806 | 22 | 13.085 | 173 | 332 | 1489 |
| Higgins J.P.T. | HEALTH | 6165 | 28 | 11.858 | 1 | 3296 | 3884 |
| Akiskal H.S. | BRAIN | 2426 | 19 | 11.941 | 132 | 572 | 1473 |
| Auwerx J. | MED | 4042 | 30 | 14.526 | 51 | 137 | 2363 |
| Dahn J.R. | ENG | 3241 | 25 | 15.593 | 29 | 288 | 3047 |
| Deary I.J. | HEALTH | 3146 | 22 | 11.769 | 69 | 652 | 1487 |
| Saver J.L. | BRAIN | 2896 | 24 | 11.251 | 129 | 417 | 1212 |
| Langdon T.G. | ENG | 2975 | 20 | 13.829 | 135 | 156 | 2688 |
| Polyak K. | MED | 3022 | 26 | 11.065 | 76 | 551 | 1339 |
| O'Keeffe M. | CHEM | 5470 | 33 | 10.958 | 49 | 562 | 709 |
| Thase M.E. | BRAIN | 3295 | 25 | 10.163 | 132 | 461 | 1110 |
| Shi Y. | MED | 1868 | 20 | 11.226 | 194 | 484 | 1532 |
| Xu H.-K. | MATH | 999 | 16 | 13.333 | 496 | 547 | 939 |
| Hecht S.S. | MED | 1597 | 15 | 11.631 | 385 | 607 | 1141 |
| Schlessinger J. | MED | 2566 | 22 | 10.371 | 246 | 318 | 1326 |
| Wing R.R. | HEALTH | 2639 | 26 | 11.861 | 54 | 696 | 1417 |
| Gabrilovich D.I. | MED | 2234 | 26 | 11.206 | 51 | 726 | 1962 |
| Monaghan J.J. | ENG | 951 | 14 | 11.583 | 656 | 813 | 919 |
| Scuseria G.E. | CHEM | 3497 | 23 | 13.951 | 68 | 299 | 1714 |
| George S.M. | PHYS | 1903 | 21 | 10.698 | 276 | 355 | 1412 |
| Cleeman J.I. | MED | 3406 | 17 | 3.808 | 1076 | 1078 | 1114 |
| Yao Y. | CS | 853 | 17 | 14.250 | 465 | 639 | 717 |
| Duarte C.M. | BIO | 2725 | 23 | 12.244 | 110 | 487 | 1098 |
| Noyori R. | CHEM | 1789 | 21 | 11.851 | 132 | 524 | 1672 |
| Bartlett J.G. | MED | 2167 | 21 | 11.008 | 222 | 454 | 1115 |
| Ferrari M. | ENG | 1835 | 19 | 11.118 | 342 | 363 | 1256 |
| Flaherty K.T. | MED | 4222 | 26 | 9.136 | 54 | 1004 | 1159 |
| Moran N.A. | BIO | 1623 | 20 | 13.860 | 159 | 463 | 1259 |
| Keasling J.D. | BIO | 2498 | 25 | 12.773 | 145 | 175 | 1882 |
| Antolini E. | ENG | 924 | 16 | 13.417 | 400 | 803 | 808 |
| Le Bihan D. | BRAIN | 1688 | 21 | 11.542 | 194 | 682 | 941 |
| Penuelas J. | BIO | 2767 | 23 | 14.669 | 31 | 753 | 1605 |
| Vallet-Regi M. | HEALTH | 2160 | 19 | 12.460 | 90 | 691 | 1672 |
| Krieger N. | HEALTH | 1093 | 15 | 13.000 | 345 | 812 | 970 |
| Somorjai G.A. | CHEM | 3252 | 25 | 11.995 | 52 | 341 | 2505 |
| Spaide R.F. | HEALTH | 1707 | 21 | 11.944 | 143 | 631 | 1256 |
| Lees A.J. | BRAIN | 5089 | 30 | 12.818 | 36 | 241 | 1910 |
| Wolpert D.M. | BRAIN | 1943 | 24 | 13.992 | 51 | 642 | 1600 |
| Iadecola C. | BRAIN | 2005 | 19 | 11.345 | 177 | 578 | 1317 |
| Badylak S.F. | MED | 2094 | 20 | 11.282 | 140 | 571 | 1490 |
| Korsmeyer S.J. | MED | 3628 | 30 | 13.882 | 52 | 139 | 2568 |
| Sampson R.J. | SOC | 1398 | 17 | 12.833 | 121 | 1127 | 1292 |
| Davis M. | BRAIN | 1677 | 19 | 12.518 | 180 | 509 | 1348 |
| Lewis K. | INFDIS | 1318 | 21 | 12.380 | 345 | 388 | 929 |
| Fombonne E. | SOC | 2079 | 24 | 10.477 | 306 | 437 | 687 |
| Leydesdorff L. | SOC | 1253 | 16 | 12.617 | 251 | 737 | 1208 |
| Poulin R. | BIO | 1399 | 15 | 12.172 | 358 | 609 | 1137 |
| Tropp J.A. | CS | 1543 | 13 | 8.650 | 393 | 1172 | 1532 |
| Yang X.-S. | CS | 1000 | 16 | 12.250 | 434 | 806 | 819 |
| Duffau H. | BRAIN | 1607 | 21 | 12.360 | 194 | 544 | 992 |
| Whittingham M.S. | ENG | 1208 | 16 | 10.950 | 515 | 567 | 1084 |
| Zur Hausen H. | MED | 1324 | 14 | 10.955 | 566 | 639 | 1048 |
| Woosley S.E. | PHYS | 1881 | 21 | 12.995 | 103 | 642 | 1233 |
| Grimes C.A. | ENG | 3328 | 30 | 13.387 | 62 | 128 | 2690 |
| Kupfer D.J. | BRAIN | 4172 | 25 | 12.678 | 51 | 237 | 2420 |
| Eichenbaum H. | BRAIN | 1432 | 19 | 13.833 | 180 | 494 | 1221 |
| Wardle J. | MED | 2701 | 22 | 13.891 | 39 | 598 | 1963 |
| Speakman J.R. | BIO | 1635 | 19 | 12.525 | 162 | 601 | 1266 |
| Jackson S.P. | MED | 2793 | 26 | 14.495 | 45 | 317 | 1949 |
| Blaabjerg F. | CS | 3562 | 27 | 14.078 | 15 | 566 | 2604 |
| Nelson J.K. | PHYS | 2671 | 24 | 13.543 | 142 | 240 | 1135 |
| Blasco M.A. | MED | 2266 | 23 | 12.693 | 162 | 274 | 1312 |
| Kuller L.H. | MED | 5220 | 31 | 12.176 | 39 | 327 | 1256 |
| Happe F. | SOC | 1491 | 21 | 12.771 | 220 | 573 | 788 |
| Thrun S. | CS | 1788 | 22 | 12.539 | 135 | 458 | 1324 |
| MacMillan D.W.C. | CHEM | 1924 | 25 | 15.900 | 128 | 132 | 1904 |
| Schrock R.R. | CHEM | 1684 | 16 | 12.819 | 280 | 524 | 1047 |
| Weigel D. | BIO | 3919 | 31 | 14.646 | 29 | 232 | 1969 |
| Brunger A.T. | BIO | 1753 | 16 | 9.991 | 241 | 845 | 1364 |
| Falkowski P.G. | BIO | 2252 | 22 | 11.678 | 83 | 534 | 1798 |
| Sun D.-W. | BIO | 1640 | 19 | 14.867 | 170 | 390 | 1155 |
| Epstein J.I. | MED | 3025 | 21 | 10.724 | 85 | 604 | 1588 |
| Halestrap A.P. | MED | 1387 | 20 | 12.547 | 207 | 540 | 1094 |
| Scherer P.E. | MED | 3107 | 27 | 14.578 | 55 | 214 | 1856 |
| Bruix J. | MED | 4993 | 30 | 10.320 | 4 | 1488 | 4101 |
| Crawley J.N. | BRAIN | 1751 | 22 | 13.740 | 176 | 310 | 1172 |
| Case D.A. | CHEM | 3250 | 22 | 11.960 | 51 | 480 | 2173 |
| Eysenbach G. | HEALTH | 928 | 15 | 12.926 | 436 | 759 | 873 |
| Granqvist C.G. | ENG | 1447 | 17 | 11.113 | 330 | 572 | 1153 |
| Laurance W.F. | BIO | 1621 | 20 | 10.900 | 198 | 738 | 1013 |
| Russell P.St.J. | CS | 2148 | 20 | 9.965 | 292 | 312 | 1545 |
| Jones D.P. | MED | 1849 | 18 | 12.755 | 231 | 422 | 1120 |
| Araujo M.B. | BIO | 2573 | 29 | 12.981 | 32 | 780 | 1183 |
| Cohen M. | MED | 3285 | 25 | 11.622 | 103 | 445 | 891 |
| Kenny D.A. | SOC | 3286 | 15 | 11.233 | 100 | 426 | 3157 |
| Hosono H. | PHYS | 3386 | 21 | 10.919 | 108 | 218 | 2933 |
| Fiehn O. | CHEM | 1742 | 22 | 10.893 | 235 | 474 | 1000 |
| Lee W.M. | MED | 1983 | 20 | 11.939 | 276 | 419 | 818 |
| Baulcombe D.C. | BIO | 2006 | 24 | 12.958 | 174 | 197 | 1562 |
| Storey J.D. | BIO | 1498 | 17 | 8.459 | 380 | 956 | 1109 |
| Goldsmith A. | CS | 1980 | 20 | 11.767 | 112 | 608 | 1512 |
| Esler M.D. | MED | 2557 | 23 | 10.843 | 70 | 660 | 1622 |
| Davidson E.A. | BIO | 1920 | 21 | 10.995 | 97 | 1005 | 1156 |
| Kramer M.S. | MED | 1725 | 18 | 11.220 | 155 | 822 | 1263 |
| Montgomery D.R. | EARTH | 1347 | 20 | 13.900 | 147 | 641 | 1004 |
| Hoegh-Guldberg O. | BIO | 2433 | 18 | 9.861 | 174 | 691 | 1323 |
| Andreae M.O. | EARTH | 2293 | 21 | 11.283 | 99 | 673 | 1330 |
| Black R.E. | MED | 3183 | 21 | 10.914 | 48 | 781 | 1939 |
| Angulo P. | MED | 1531 | 19 | 9.659 | 353 | 601 | 1045 |
| Belsky J. | SOC | 1598 | 16 | 10.787 | 237 | 918 | 1082 |
